# Prediction of Yangtze River streamflow based on deep learning neural network with El Niño–Southern Oscillation

**DOI:** 10.1038/s41598-021-90964-3

**Published:** 2021-06-03

**Authors:** Si Ha, Darong Liu, Lin Mu

**Affiliations:** 1 College of Marine Science and Technology, China University of Geosciences, Wuhan, 430074 China; 2 College of Life Sciences and Oceanography, Shenzhen University, Shenzhen, 518060 China

**Keywords:** Computer science, Hydrology

## Abstract

Accurate long-term streamflow and flood forecasting have always been an important research direction in hydrology research. Nowadays, climate change, floods, and other anomalies occurring more and more frequently and bringing great losses to society. The prediction of streamflow, especially flood prediction, is important for disaster prevention. Current hydrological models based on physical mechanisms can give accurate predictions of streamflow, but the effective prediction period is only about 1 month in advance, which is too short for decision making. The artificial neural network (ANN) has great potential for predicting runoff and is not only good at handling non-linear data but can also make long-period forecasts. However, most of ANN models are unstable in their predictions when faced with raw flow data, and have excessive errors in predicting extreme flows. Previous studies have shown a link between the El Niño–Southern Oscillation (ENSO) and the streamflow of the Yangtze River. In this paper, we use ENSO and the monthly streamflow data of the Yangtze River from 1952 to 2018 to predict the monthly streamflow of the Yangtze River in two extreme flood years and a small flood year by using deep neural networks. In this paper, three deep neural network frameworks are used: stacked long short-term memory, Conv long short-term memory encoder–decoder long short-term memory and Conv long short-term memory encoder–decoder gate recurrent unit. The results show that the use of ConvLSTM improves the stability of the model and increases the accuracy of the flood prediction. Besides, the introduction of ENSO to the experimental data resulted in a more accurate prediction of the time of the occurrence of flood peaks and flood flows. Furthermore, the best results were obtained on the convolutional long short-term memory + encoder–decoder gate recurrent unit model.

## Introduction

The Yangtze River is one of the most important rivers in China, with a large, densely populated, and economically developed river basin. Flooding in the Yangtze River is of great concern to people, and China has invested heavily in flood prevention. However, thousands of people still died in several major floods in the past three decades, and the average direct loss is more than 100 billion RMB per year^1^. Yangtze River streamflow forecasting plays an important role in flood prevention and post-disaster relief, as well as in integrated water resources development and utilization, scientific management, and optimal scheduling. Because many factors affect the streamflow of the Yangtze River^2^, researchers have used various methods to predict the streamflow of the Yangtze River over the years to obtain valuable prediction data.

Runoff is a natural signal, a complex non-linear time series that is simultaneously influenced by a variety of factors such as rainfall in the basin, the degree of erosion in the basin, atmospheric circulation, and urban and rural water use. Different methods of flow prediction have been proposed by researchers for predicting runoff. These methods can be divided into short-term prediction methods, dealing with prediction times of hours^3,4^ to days^5–7^, and long-term prediction methods, dealing with scales of weeks^8^, months^7,9^, and even years^10^. These methods can also be divided according to the type of model employed: hydrological models based on physical mechanisms and data-driven models based on data analysis. Hydrological models include the Soil and Water Assessment Tool (SWAT), Top Model^11^, and the Xinanjiang model^12^. These models simulate the variability and transport of elements such as water quantity and quality in a region by collecting spatial and hydrological information about the river basin to obtain a prediction of river streamflow. This class of models has been widely validated and applied to achieve river streamflow prediction. Among the data-driven models, there are traditional black box time series models such as auto-regressive, moving average, auto-regressive moving average, auto-regressive integrated moving average, and auto-regressive integrated moving average with exogenous input models[^13–16^. These models look for patterns by linearly decomposing the streamflow data, and thus perform well when the data has periodic features; however, they perform poorly in the face of complex hydrological data. Data-driven models also include artificial intelligence (AI) models, which are good at dealing with nonlinear data and can find patterns in noise; therefore, AI-based models perform well when dealing with hydrological problems. Such models include artificial neural networks (ANNs)^17^], the support vector machine (SVM)^18^, backpropagation (BP), fuzzy sets^19^, evolutionary computation (such as evolution strategies)^20^, and wavelet conjunction models^21^ etc. The SVM model performs better than the gene expression programming and M5 model trees^22^. Although ANNs perform with average accuracy compared to numerical statistical methods^23^, they have great potential for development. ANNs were inspired by the structure of biological neurons^17^ and simulate these biological neurons, essentially constructing a mapping with a large number of parameters to fit the mapping between actual observed data and predicted data. This configuration of ANNs makes them excellent at handling nonlinear data with implicit patterns. The features of hydrological data are well matched to ANNs, and thus they perform well when dealing with hydrological data. Previous studies have also found LSTM to be more accurate than BP and SVM for daily streamflow prediction, but to overfit^24^.

In recent years, both physics-based hydrological models and ANNs have been studied and applied in the processing of hydrological data. The physics-based hydrological model requires a large amount of existing hydrogeological data to construct a hydrogeological model of the study area, and even uses future hydrological data, such as future rainfall data. Hydrological models based on physical mechanisms have the following drawbacks: (1) they do not yield valid results in data-poor areas; (2) they require the use of high-precision rainfall predictions to support calculations; (3) although they are highly accurate in predicting normal streamflow, they are less sensitive to anomalies (e.g., floods and droughts) and less accurate in predicting extreme weather; (4) short-term forecasts (hours, days) are highly accurate, while long-term forecasts are less accurate. ANNs solve the streamflow prediction problem differently, and their structure can well match streamflow data. Models such as long short-term memory (LSTM) models are excellent in dealing with the time series problem, and have been widely used in natural language processing^25,26^, image recognition^27^, automated driving^27^, and time series prediction^28,29^. ANN models have the following advantages over hydrological models in predicting streamflow: (1) they require less data, and most studies have achieved good results using only streamflow data; (2) they predict on many time scales, such as daily, weekly, monthly, and yearly time scales; (3) they are better at capturing hidden features in historical data and more accurately predict outliers^30^. The ANN can be combined with similar numerical statistical analysis methods such as moving average (MA) and singular spectrum analysis (SSA) for hydrological data prediction. Pre-processing hydrological data with MA and SSA help ANNs to learn patterns in the data. This increases the generalization capability of the model^31^. Among the preprocessing methods besides MA and SSA some methods use empirical mode decomposition to do preprocessing, combined with deep learning algorithms to study the prediction of river flow and El Nino^32,33^. However, it is also pointed out in these articles that the results are not accurate when machine learning models are trained directly using the original data.

In recent years, the main focus of ANNs has been to improve the structure of models so that they can better exploit implicit connections in the data, and discover connections with longer time horizons, thus improving prediction performance. Many ANNs have emerged to offer more and better solutions to the time series processing problem. These methods include LSTM, which is good at dealing with continuous time series; convolutional neural networks (CNNs), which are good at dealing with spatially characterized data like through satellite imagery identify disaster areas^34^; also there are convolutional long short term memory (Conv LSTM), gate recurrent unit (GRU) and encoder–decoder structure.

The accuracy of river streamflow prediction from the perspective of training data can be improved not only by exploring correlations in streamflow history data but also by including streamflow correlations other than streamflow data in the training, thereby improving the prediction results. Previous studies have found relationships between river streamflow and various data, such as precipitation, sea surface temperature, wetness, sea level pressure, evaporation, the El Niño–Southern Oscillation (ENSO), and the East Asian Summer Monsoon (EASM). Nalley et al. revealed a relationship between streamflow ENSO, the North Atlantic Oscillation (NAO) and the Pacific Decadal Oscillation (PDO)^35^, while Wei et al. found a relationship between the EASM and ENSO and the Yangtze River’s streamflow rate. Moreover, it was found that weak EASMs and ENSOs can lead to extreme floods, while strong EASMs and ENSOs can lead to extreme droughts^36^.

Timo et al. studied the temporal and spatial effects of ENSO on precipitation fluctuations and flood occurrence in the Mekong River Basin, and their results showed that El Niño was negatively correlated with flooding while La Niña was positively correlated with flooding. Meanwhile, the average annual flood cycle in La Niña increased by 1 month compared to El Niño years, and the precipitation and streamflow anomalies during El Niño were found to be larger than those during La Niña^37^. In a study investigating the link between streamflow volume and the ENSO in the Yangtze River, Zhang Zizhan et al. used GRACE data to investigate the link between terrestrial water storage and ENSO in the Yangtze River basin^38^. The upstream streamflow and ENSO phases are inversely correlated while the downstream streamflow and ENSO phases are positively correlated^39^. Furthermore, Jiang et al. point out La Niña is strongly associated with drought events and El Niño related to floods in the middle and lower Yangtze River basin, while the opposite is true in the upper Yangtze River basin^40^. From the above study, it can be seen that there is a correlation between ENSO values and numerous values, especially a significant correlation with floods. In particular, ENSO values are remotely correlated with values of regional precipitation and streamflow in China. Therefore ENSO values are more suitable for flood prediction than rainfall and other data.

The types of data used for solving streamflow prediction problems with artificial intelligence include streamflow, precipitation, sea surface temperature, wetness, sea level pressure, and evaporation. As Sharma compared the differences between the adaptive neuro-fuzzy inference system and the Loading Simulation Program in C++ model using these types of data and found that the two methods produced similar results^41^. Typically, the data used for streamflow prediction using ANNs are streamflow, evaporation, and precipitation; ENSO data has not been used^3,7,42^. To investigate whether the introduction of ENSO values into the streamflow prediction problem will help improve the accuracy of streamflow prediction, the present paper adds ENSO values to the training data of several better-performing and widely used ANN models. We also made a new improvement to the ANN,by using ConvLSTM as the encoder in encoder–decoder structure and compared it with stacked LSTM in terms of accuracy and fitting ability of flood prediction.

## Methodology

### Long short-term memory

Long short-term memory (LSTM) was proposed by Sepp Hochreiter et al. in 1997^43^. It is an algorithm based on the recurrent neural network (RNN). LSTM solves the vanishing gradient problem by introducing three thresholds and two memory states^44^.

1 $$\begin{array}{c}{i}_{t}=\sigma \left({W}_{i}\left[{h}_{t-1}, {x}_{t}\right]+{b}_{i}\right)\end{array}$$

2 $$\begin{array}{c}{f}_{t}=\sigma \left({W}_{f}\left[{h}_{t-1},{x}_{t}\right]+{b}_{f}\right)\end{array}$$

3 $$\begin{array}{c}{o}_{t}=\sigma \left({W}_{o}\left[{h}_{t-1},{x}_{t}\right]+{b}_{o}\right)\end{array}$$

4 $$\begin{array}{c}{h}_{t}={o}_{t}\circ \mathrm{tanh}\left({C}_{t}\right)\end{array}$$

5 $$\begin{array}{c}{C}_{t}={f}_{t}\circ {C}_{t-1}+{i}_{t}\circ {\tilde{C }}_{t}\end{array}$$

6 $$\begin{array}{c}{\tilde{C }}_{t}=\mathrm{tanh}\left({W}_{c}\left[{h}_{t-1}, {x}_{t}\right]+{b}_{C}\right)\end{array}$$

LSTM consists of three gates: input gate ${i}_{ t}$; forget gate ${f}_{ t}$; and output gate ${o}_{ t}$. The two mnemonic states are the cell state ${C}_{ t}$ and candidate state ${\tilde{C }}_{ t}$. The formulas used by LSTM are Eqs. (1)–(6). ${W}_{ i}$, ${W}_{ f}$, and ${W}_{ o}$ comprise the matrix of parameters to be trained. ${b}_{ i}$, ${b}_{ f}$, and ${b}_{ o}$ are the biases to be trained. ${x}_{ t}$ is the entered data. ${ h}_{ t-1}$ is the result of the last moment of memory. ${h}_{t}$ represents the short term memory and the ${C}_{ t}$ cell state represents the long term memory.

**Figure 1:**
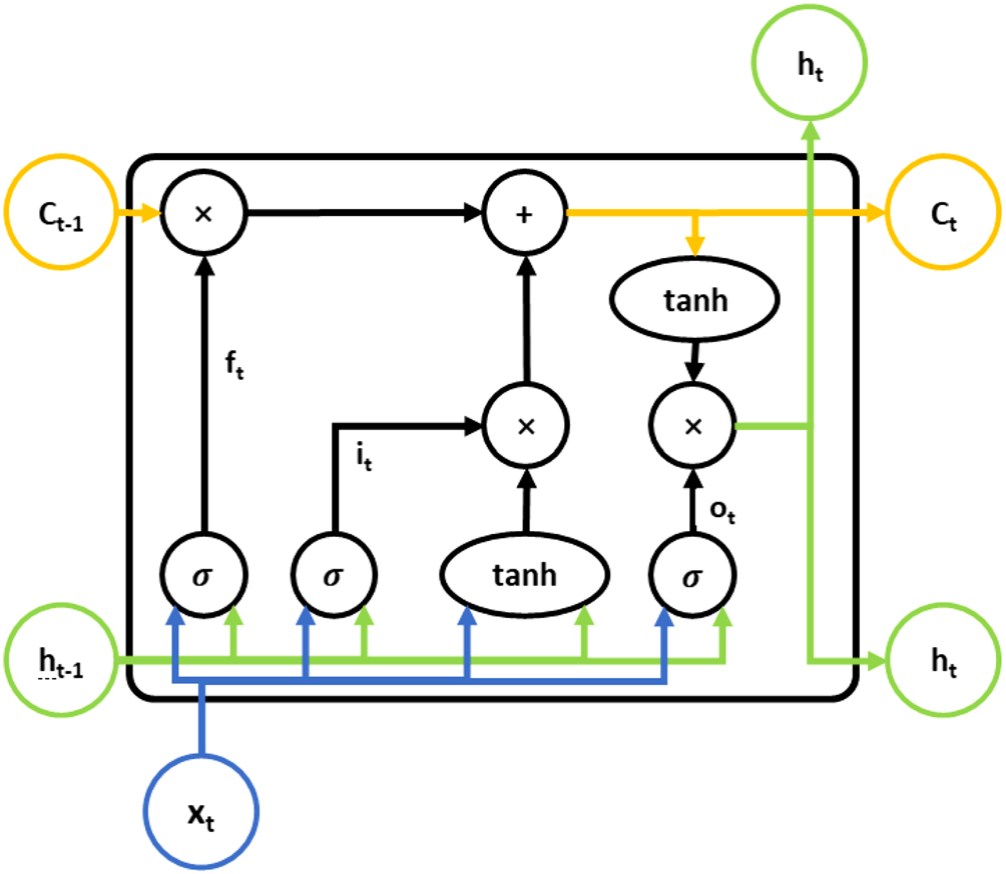
Networks of the LSTM unit.

The formula for the input gate is (1), the formula for the forget gate is (2), and the formula for the output gate is (3). The tanh activation function limits the output to between − 1 and 1 and can be replaced by other activation functions. The three gates multiply the input data and the memory of the previous moment and output. Equation (4) is the formula for memory, which is the result of multiplying the output data from the current output gate with the cell state that has undergone the tanh function; the memory represents the short term memory resulting from the action of the output and the long term memory. The cell state represents the long term memory and is calculated as in (5) by multiplying the cell state at the previous moment through the forget gate by the candidate state. The candidate state represents the information to be deposited in the cell state, and is calculated as in (6); it is the result of the action of the current input data and the output data from the previous moment. Figure 1 shows the structure of an LSTM memory unit.

**Figure 2:**
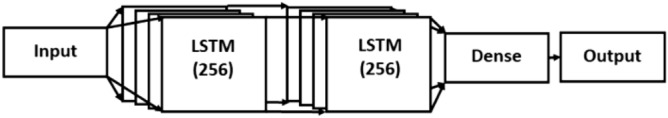
Stacked LSTM structure.

In this paper, the LSTM model is used in stacked LSTM and convolutional LSTM encoder–decoder LSTM. Figure 2 illustrates the stacked LSTM used for the experiments in this paper. The body of the model is a two-layer LSTM containing 256 memory cells. The output passes through the dense layer. Three layers of LSTMs are used as a decoder in convolutional LSTM encoder–decoder LSTM to decode the encoded vectors and output them through the Dense layer.

### Gate recurrent unit

The gate recurrent unit (GRU) was proposed by Cho et al. in 2014^45^ to solve the vanishing gradient problem in RNN networks. The GRU can be regarded as the deformation of LSTM. It has fewer parameters than LSTM and can produce the same excellent results as LSTM in some cases. The features of the GRU make it possible to shorten the computation time without affecting the prediction performance and even produce better results, thus making it a frequently used model in machine learning^46^.

**Figure 3:**
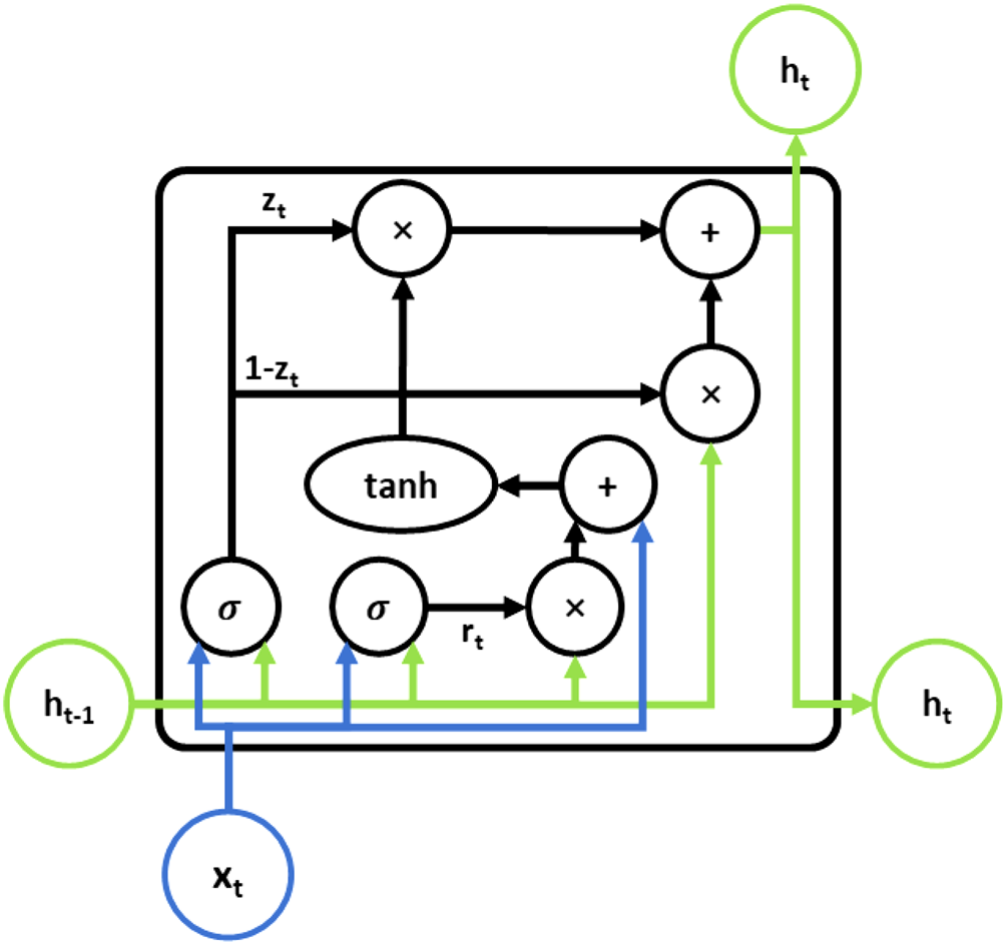
Networks of GRU unit.

The GRU is similar in principle to LSTM, with an update gate (7), a reset gate (8), a memory (9), and a candidate hidden layer (10). $\sigma$ is the Sigmoid function, which limits the output to the range 0 to 1, and the tanh function limits the output to the range − 1 to 1. ${W}_{ z}$, ${W}_{ r}$ and $W$ is the parameter matrix. Both the update gate and the reset gate calculate the memory of the current input and the previous moment. The updates gate determines the update of the memory and controls how much of the previous moment's memory and the current input data can be retained in the current memory. The reset gate also determines the update of the memory by controlling the candidate state but controls how much of the information from the previous memory is forgotten. The candidate hidden layer represents the memory formed at the current moment. Figure 3 shows the structure of a GRU memory unit.

7 $$\begin{array}{c}{z}_{t}=\sigma \left({W}_{z}[{h}_{t-1},{x}_{t}]\right)\end{array}$$

8 $$\begin{array}{c}{r}_{t}=\sigma \left({W}_{r}[{h}_{t-1},{x}_{t}]\right)\end{array}$$

9 $$\begin{array}{c}{h}_{t}=\left(1-{z}_{t}\right)\circ {h}_{t-1}+{z}_{t}\circ {\tilde{h }}_{t}\end{array}$$

10 $$\begin{array}{c}{\tilde{h }}_{t}=\mathrm{tanh}\left(W[{r}_{t}\circ {h}_{t-1},{x}_{t}]\right)\end{array}$$

In the experiments set up in this paper, the convolutional LSTM encoder–decoder GRU (Conv LSTM encoder–decoder GRU) uses a three-layer GRU as the decoder structure to decode the encoded vectors and output them through the dense layer.

### Convolutional LSTM network

**Figure 4:**
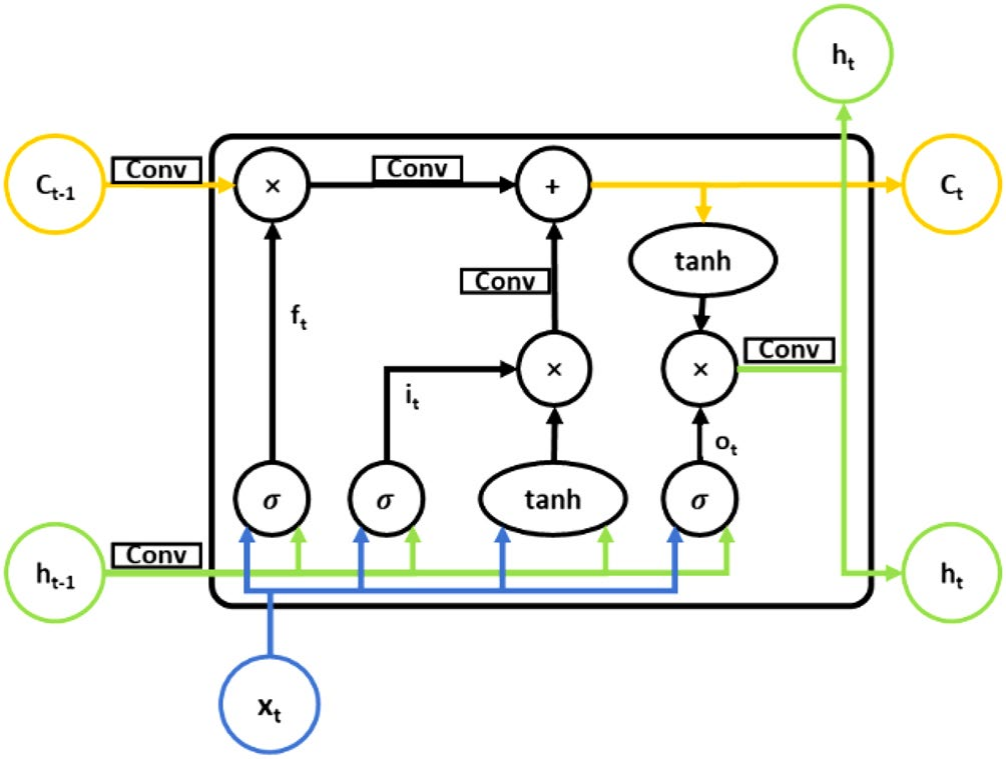
Networks of Conv LSTM unit.

The convolutional LSTM network (Conv LSTM) was proposed by Shi Xingjian et al. in 2015^47^. In the past, LSTM was used as the encoder layer when building encoder–decoder models; however, LSTM has no special design for spatial–temporal sequences and uses full connections between layers to transform data. Meanwhile, Conv LSTM uses convolution instead of full connections to transform data. Conv LSTM has roughly the same formula as LSTM, using formulas (11)–(16), but the * stands for convolution instead of a full-connection operation; otherwise, the meaning and function of each formula is as in the LSTM and described above. According to Shi Xingjian's article, a larger kernel can perceive features with larger spatial variation in the data while a smaller kernel can perceive features with a small spatial variation. Figure 4 shows the structure of a Conv LSTM memory unit. Compared to the fully connected LSTM there is a lot of redundancy in the computation and it does not take spatial correlation into account very well. The ConvLSTM with the addition of convolutional computation has better results in obtaining spatio–temporal relationships. This makes ConvLSTM more suitable than LSTM for predicting hydrological data.

11 $$\begin{array}{c}{i}_{t}=\sigma \left({W}_{i}*\left[{h}_{t-1}, {x}_{t}\right]+{b}_{i}\right)\end{array}$$

12 $$\begin{array}{c}{f}_{t}=\sigma \left({W}_{f}*\left[{h}_{t-1},{x}_{t}\right]+{b}_{f}\right)\end{array}$$

13 $$\begin{array}{c}{o}_{t}=\sigma \left({W}_{o}*\left[{h}_{t-1},{x}_{t}\right]+{b}_{o}\right)\end{array}$$

14 $$\begin{array}{c}{h}_{t}={o}_{t}\circ \mathrm{tanh}\left({C}_{t}\right)\end{array}$$

15 $$\begin{array}{c}{C}_{t}={f}_{t}\circ {C}_{t-1}+{i}_{t}\circ {\tilde{C }}_{t}\end{array}$$

16 $$\begin{array}{c}{\tilde{C }}_{t}=\mathrm{tanh}\left({W}_{c}*\left[{h}_{t-1}, {x}_{t}\right]+{b}_{C}\right)\end{array}$$

Conv LSTM was originally developed to process a series of radar wave images and extract the motion of clouds according to the time series of radar wave images, thus giving accurate short-term predictions. In this paper, the streamflow data and ENSO data are 1-dimensional data that change with time. When using Conv LSTM, the time series are first grouped according to different periods, and then the grouped 1-dimensional data are treated as special 2-dimensional data, and the streamflow data and ENSO data are composed of a sequence with two channels fed into the Conv LSTM network. After the above procedure, the convolutional kernel extracts the feature information from the time series as spatial features, thus increasing the accuracy of the prediction.

### Conv LSTM encoder–decoder RNN

**Figure 5:**
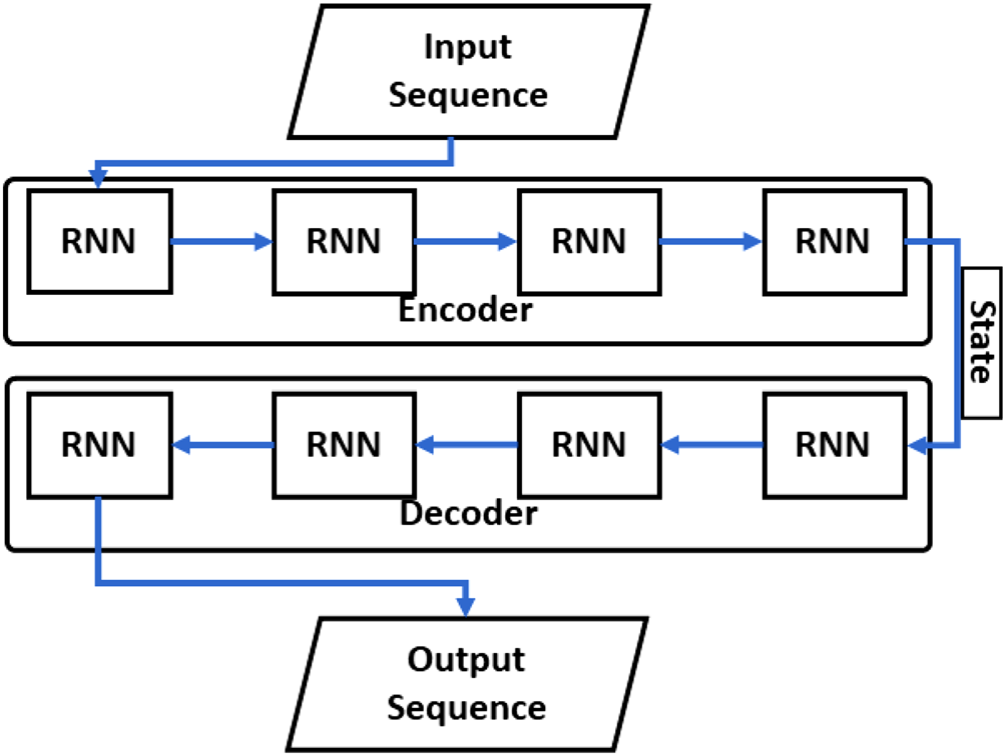
Architecture of encoder–decoder.

The encoder–decoder model was proposed by Ilya Sutskever et al. to solve the problem of needing a sufficient amount of annotation data for training traditional deep neural networks (DNNs)^48^. The encoder–decoder structure is shown in Fig. 5. The encoder encodes the input field into a vector and the decoder decodes the encoded vector into the output field. Ilya Sutskever et al. found encoder–decoder structure constructed by the LSTM model handles the translation results similar to the best translation results at that time. Therefore, the encoder–decoder structure is often used to handle the sequence-to-sequence problem. The encoder–decoder model has one feature when dealing with the sequence-to-sequence (seq2seq) problem: it is sensitive to the order of the input sequences, which means that encoder–decoder may perform well in dealing with the time series problem. Compared to other networks that deal directly with the seq2seq problem, the addition of a decoder as a hidden layer increases the complexity of the model and also brings an improvement in prediction accuracy. Since streamflow prediction using time series data consisting of streamflow and ENSO values to predict future streamflow data can also be used as a sequence-to-sequence problem, the encoder–decoder structure is chosen for our experiments.

**Figure 6:**
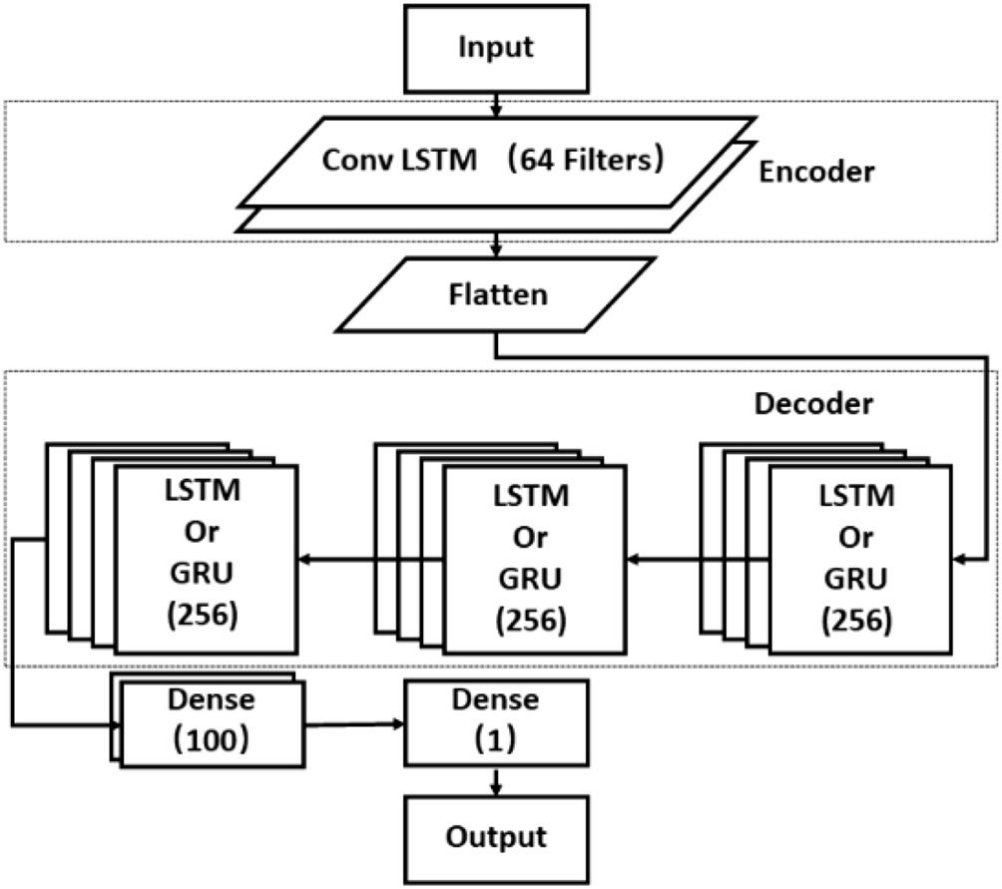
Conv LSTM encoder–decoder.

The Conv LSTM encoder–decoder RNN used in this paper uses encoder–decoder as the model framework (Fig. 6). The selection of the convolution kernel parameters here is based on the empirical choice of the parameter settings that work relatively well for the model. There are also search methods such as grid search for the selection of model parameters. This type of search method is used to find the best combination of parameters by trying different parameters. The aim is to get the best results for the model on the current data set. However, this study attempts to improve the model results by changing the model structure and therefore uses the parameter configurations that work better empirically. Although the parameters are not necessarily the best results, they can reflect the differences in performance due to different model structures. The encoder uses a Conv LSTM with 64 convolutional kernels, and the size of the convolutional kernel is (n, 3), where n is the number of training data feature values. n = 1 when the data is only streamflow data, and n = 2 when the training data contains both flow data and ENSO data. The step size of the extracted time-series features increases when the convolution kernel becomes large, while the performance of the extracted time features is close to that of an ordinary LSTM when the size of the convolutional kernel is too small. Therefore, the size of the convolution kernel is chosen to be 3. The encoder output is transformed into a one-dimensional feature vector through the flatten layer and then fed into the decoder, which uses a three-layer LSTM or GRU with 256 memory units, and finally, the decoder data are transformed into a prediction output through the dense layer. The GRU and LSTM should not be over-stacked in terms of the number of layers. Since the GRU and LSTM are structured to solve the problem of gradient vanishing between each memory, without considering the gradient vanishing between layers, too much stacking of the LSTM and GRU will make the model less effective. At the same time, as the number of stacked layers increases, the memory cost becomes higher and the computation time increases. The GRU is more suitable for simple time-series problems when the number of stacked layers is small. For better prediction results, a three-layer GRU is used here as the decoder structure. The number of memory units in the LSTM and GRU directly boosts the number of parameters in the RNN network. Increasing the number of memory units increases the fitting ability of the model. Empirically, the larger the number of memory units, the smaller the improvement in the effectiveness of the model, as well as the slow and underfitting of the model. The choice of using 128 or 256 is already a good fit and is fast enough. The structure combines multiple models while inheriting the advantages of each model. The Conv LSTM as an encoder has excellent temporal feature extraction capability and can sense the change in data over time, while the LSTM and GRU as a decoder have similar time series processing capability, and the GRU simplifies the number of parameters compared to the LSTM and saves computational resources.

## Experiment

### Study area and data

The Yangtze River is the most important water system in China and the fifth largest in the world in terms of streamflow volume. The source region is in the alpine zone with 300–400 mm precipitation; the upper reaches are mostly in the sub-humid zone with 400–800 mm precipitation, and the middle and lower reaches are in the humid zone with 800–1600 mm precipitation. The Yangtze River basin, the most flooded and severe basin in China, is also clearly influenced by monsoonal rainfall. There is a strong link with the El Niño event^49^. The middle and lower reaches of the Yangtze River are the areas with the most severe flooding, especially the area between the confluence of the Yangtze and Han rivers and Datong^50^; the floods that occurred in 1998 and 2018 caused great economic losses in the Yangtze River basin.

**Figure 7:**
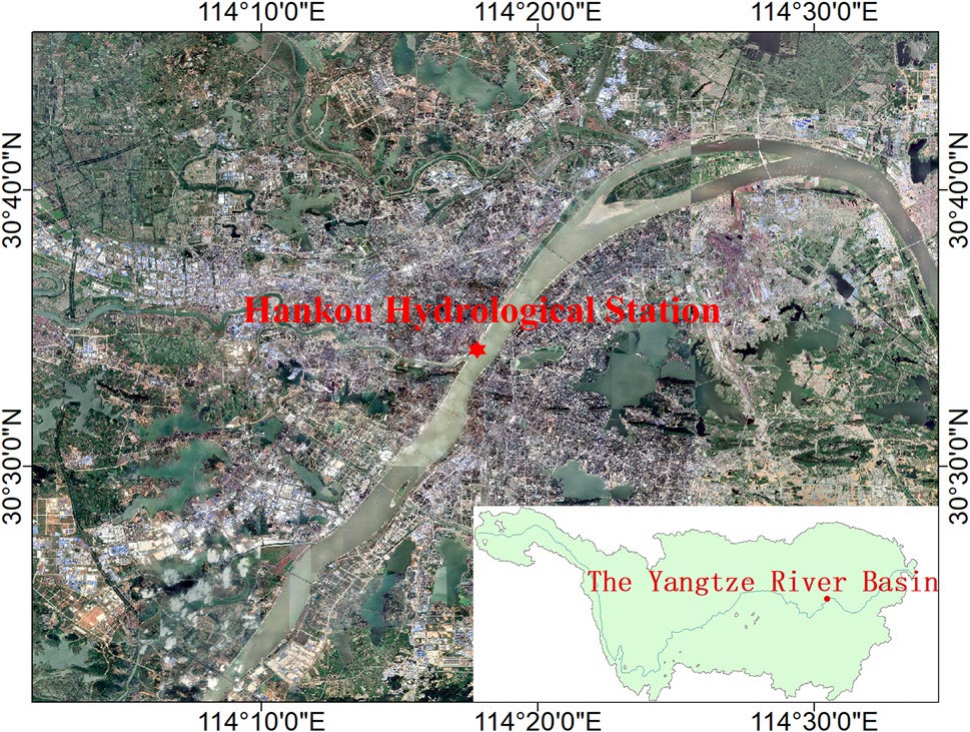
The location of the Yangtze River basin and Hankou Hydrological Station in Wuhan.

**Figure 8:**
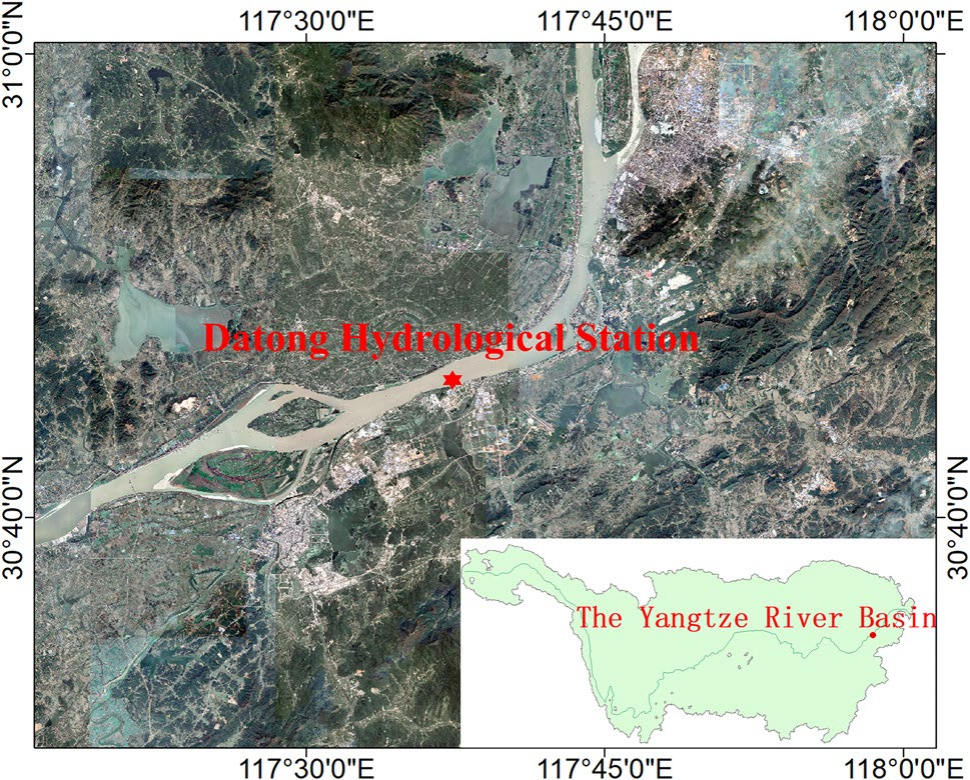
The location of the Yangtze River basin and Datong Hydrological Station in Chi Zhou.

In this experiment, we use streamflow data from the Hankou and Datong hydrological stations. The streamflow data are the monthly streamflow data of the Yangtze River from January 1952 to December 2016 recorded at the Hankou and Datong hydrological stations. The Hankou hydrological station is located in the middle reaches of the Yangtze River at the confluence of the Han and Yangtze rivers (Fig. 7) and controls a watershed area of 1,488,000 km^2^. The Datong hydrological station is located in the lower reaches of the Yangtze River, at the upper end of the Chaohe section of the Yangtze River (Fig. 8) and is the main control station for the streamflow of the main stem of the Yangtze River, with a control basin area of 1.705 million km^2^. The prediction time intervals are January–December 1998 and January–December 2016. In 1998, the second basin-wide flooding occurred in the Yangtze River basin and was characterized by high volume, prolonged flooding, and severe coastal flooding^51^. The average monthly water level at Hankou and Datong stations in April was a record high, and the average monthly water level at Hankou and Datong stations in June was about 2 m higher than that of the same period in history^52^. The model performance is verified by predicting these two abnormal years.

As a large-scale ocean–atmosphere phenomenon in the tropical Pacific, the El Niño–Southern Oscillation (ENSO) is the most important source of interannual climate variability. El Niño represents oceanic warming in the tropical central-eastern Pacific and La Niña is the opposite. Southern oscillation is characterized by a seesaw of sea level pressure between the tropical western and eastern Pacific. The occurrence of ENSO is accompanied by a series of high-intensity climate anomalies. ENSO events influence the ecosystem, agriculture, and extreme weather of a region.

**Figure 9:**
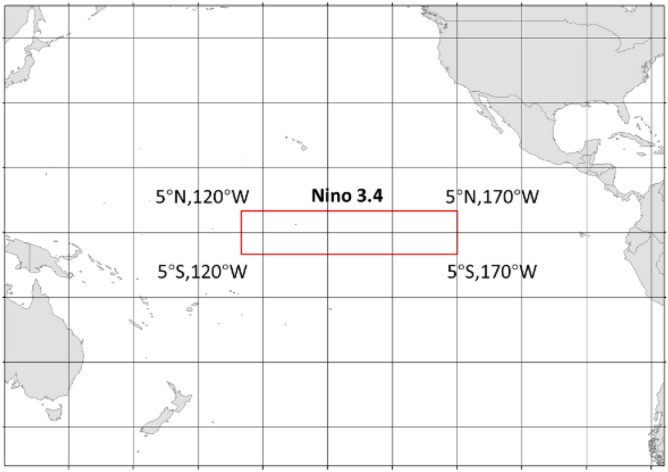
The Nino 3.4 area.

Generally, ENSO can be described by the Niño index. That is a 3-month running mean of sea surface temperature anomalies in the Niño 3.4 region (5° N–5° S, 120° W–170° W). Figure 9 shows the Niño 3.4 area. The data collected here are used as ENSO values and for the training of the model.

### Normalization

Before the data are fed into the neural network, the streamflow data and ENSO data from Hankou and Datong stations are normalized using Eq. (1) for fast convergence and stability of the model during training. ${Z}_{ i}$ is the normalized data, ranging from 0 to 1, ${X}_{ i}$ is each data, max(X) and min(X) are the maximum and minimum values of the data respectively.

17 $$\begin{array}{c}{Z}_{i}=\frac{{X}_{i}-\mathrm{min}\left(X\right)}{\mathrm{max}\left(X\right)-\mathrm{min}\left(X\right)}\end{array}$$

### Neural network construction

In this paper, three neural networks are used: the LSTM model, the GRU model, and the Conv LSTM model. These three neural networks are used to build three model frameworks: stacked LSTM, Conv LSTM encoder–decoder LSTM model and Conv LSTM encoder–decoder GRU model. These three model frameworks, ranging from simple to complex, are used to compare the effects of different numbers of eigenvalues of training data on the accuracy of flood prediction. The training data are used for both the 1-feature training data and the 2-features training data: 1-feature data contains only monthly streamflow data, while 2-feature data contains monthly streamflow data and ENSO values. The period of the training set segmentation cycle determines the "field of view" of the model, which represents the range of data that can be seen in a single read. Although the model in this study can remember previous data, the process of forming a memory is affected by the length of the input data segment, and too small a division period will result in slower training and more unstable models. Longer periods result in shorter training times for the model, but relatively less variation in flooding over longer data series, resulting in poorer model performance. We divide the training sets into four cycles: 6 months minimum prediction periods (6 m-min-pd), 12 m-min-pd, 18 m-min-pd, and 24 m-min-pd. We then compare the effects of the different number of features on the accuracy of flood prediction by assessing the training results produced under these four cycles. The “N” m-min-pd indicates a set of “N” months of data for training.

### Performance evalution

After the model has completed its predictions outputting data in the range 0–1, the normalized data ${Z}_{ i}$ need to be reduced to the original size data ${X}_{ i}$ using Eq. (18) when performing the evaluation.

18 $$\begin{array}{c}{X}_{i}={Z}_{i}*\left(\mathrm{max}\left(X\right)-\mathrm{min}\left(X\right)\right)+\mathrm{min}\left(X\right)\end{array}$$

To measure the difference between the true and predicted values, we used the following four statistics.

The root mean square error (RMSE) is defined as Eq. (19).

19 $$\begin{array}{c}RMSE=\sqrt{\frac{1}{m}\sum_{i=1}^{m}{\left({y}_{i}-{\widehat{y}}_{i}\right)}^{2}}\end{array}$$

The RMSE is the inverse square of the mean square error. The inverse square method reduces the MSE by an order of magnitude so that the scale of the result is the same as that of the original data, making it possible to compare the results more intuitively. When evaluating data that are expected to follow a Gaussian distribution, the RMSE is more suitable than the MAE to reflect the model performance^53^.

The coefficient of determination (${R}^{2}$) is defined as Eq. (20).

20 $$\begin{array}{c}{R}^{2}=1-\frac{\sum_{i=1}^{n}{\left({y}_{i}-\widehat{{y}_{\imath}}\right)}^{2}}{\sum_{i=1}^{n}{\left({y}_{i}-\overline{{y }_{\imath}}\right)}^{2}}\end{array}$$

The coefficient of determination reflects what percentage of the fluctuations in the predicted value ${y}_{i}$ can be explained by the fluctuations in the observed values^54^. The decision coefficient takes values in the range − ∞ to 1. ${R}^{2}$ close to 1 indicates that the fluctuations in the predicted values are well explained by the fluctuations in the observed values. On the contrary, a smaller ${R}^{2}$ value means that the fluctuations in the predicted values are less linearly related to the observed values and the predicted values are not well explained by the observed values.

Willmott's Index of agreement (WI) is as shown in Eq. (21).

21 $$\begin{array}{c}WI=1-\left[\frac{\sum_{i=1}^{n}{\left({y}_{i}-\widehat{{y}_{\imath}}\right)}^{2}}{\sum_{i=1}^{n}{\left(\left|\widehat{{y}_{\imath}}-\overline{{y }_{\imath}}\right|+\left|{y}_{i}-\overline{{y }_{\imath}}\right|\right)}^{2}}\right]\end{array}$$

WI is often used in the measurement of hydrological data. It is dimensionless, and is bounded by − ∞ and 1.0. It also quite flexible and is suitable for a wide range of model performance problems. In general, it is more related to model accuracy than are other indices. It was proposed by Nash and Sutcliffe in 1970, Watterson in 1996, Legates and McCabe’s in 1999, Mielke and Berry in 2001 and refined by Willmott in 2011^55^.

Legates–McCabe's Index (LMI) is written as in Eq. (22).

It is not oversensitive to extreme values and can reflect additive and proportional between model predictions and observations. The index is better suited as a complement to assessment instruments than other correlation-based assessment instruments. It is also dimensionless, bounded by 0 and 1.0. The higher the LMI value, the better the fitting effect of the model^56^.

22 $$\begin{array}{c}LMI=1-\left[\frac{\sum_{i=1}^{n}\left|\widehat{{y}_{\imath}}-{y}_{i}\right|}{\sum_{i=1}^{n}\left|{y}_{i}-\overline{{y }_{\imath}}\right|}\right]\end{array}$$

Among all the equations, where n represents the number of data pairs, ${y}_{i}$ is the observed values, $\widehat{{y}_{\imath}}$ represents the forecasted value and ${y}_{i}$ represents the mean of observed values.

## Results

**Figure 10:**
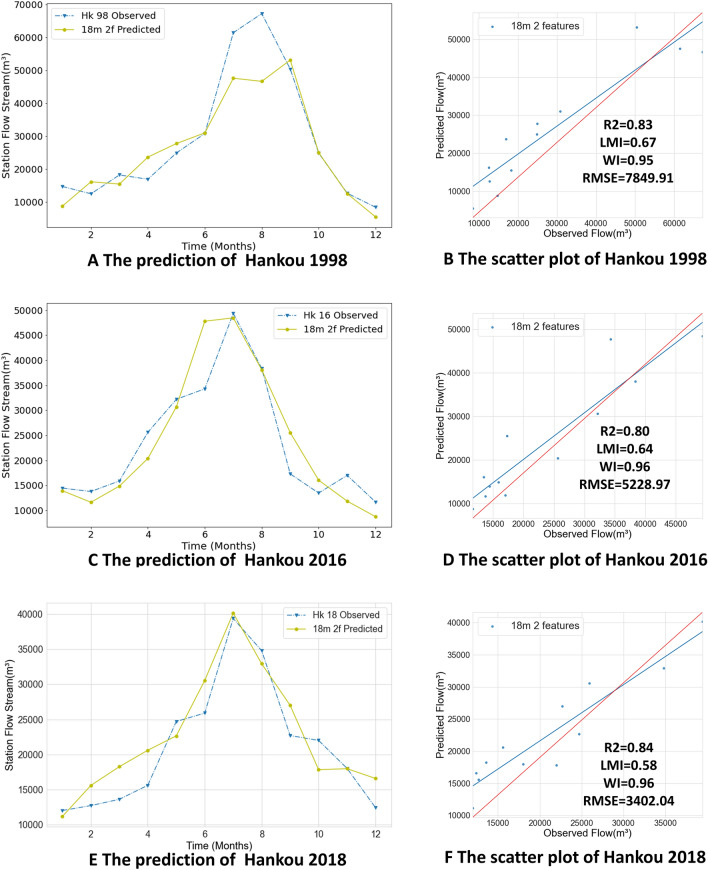
The result of Hankou Station. (**A, C, E**) Line plots of predicted and observed values. The blue dashed line represents the observed values. The green solid line are predicted values. (**B, D, F**) Scatter plots of predicted values.

**Figure 11:**
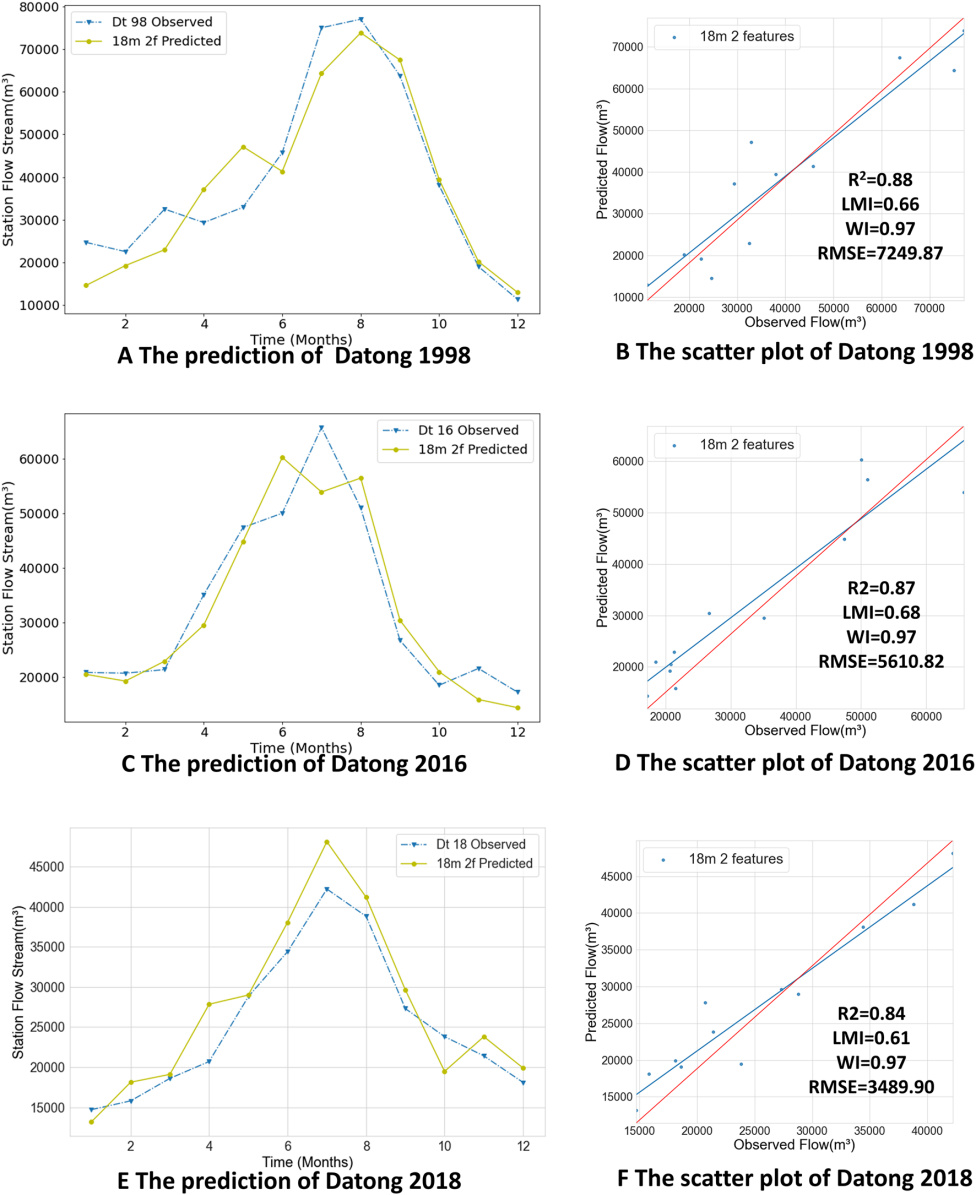
The result of Datong station. (**A, C, E**) Line plots of predicted and observed values. The blue dashed line represents the observed values. The green solid line are predicted values. (**B, D, F**) Scatter plots of predicted values.

As mentioned above, the monthly streamflow forecasts of the Yangtze River have important reference value for flood prevention, and the trained model needs to provide accurate forecasts not only in normal months but also relatively accurate forecasts of peak flows. In our experiment, the monthly streamflow forecasts of the Hankou and Datong stations, two important control stations in the middle and lower reaches of the Yangtze River, are made for the years 1998, 2016 and 2018 using Yangtze River monthly streamflow data and ENSO values. The 1998 flood was a 100-year return period flood and is classified as a very large flood, while the 2016 and 2018 floods are representative of small and medium-sized floods respectively. The dataset was split using 18-month groups and fed into the Conv LSTM encoder–decoder GRU model for prediction. Figure 10 shows the prediction results for 1998, 2016 and 2018 for the Hankou station, and Fig. 11 shows the prediction results for 1998, 2016, and 2018 for the Datong station.

The flooding trends of the Yangtze River in the past years show that flooding usually begins to converge in the middle reaches of the Yangtze River, with flood peaks in the middle and lower reaches of the river posing a great threat. As an important control station of the middle reaches of the Yangtze River, the Hankou station reflects the overall level of flooding in the middle reaches of the Yangtze River; this station’s streamflow is also an important indicator for flood control in Wuhan as well as downstream areas. The 1998 flood streamflow was huge, reaching a peak flow of nearly 70,000 m^3^ in August, and the streamflow in July nearly as high. The Hankou 1998 forecast has large deviations for July and August: 13,837.25 m^3^ between July’s forecasted and observed values, and 20,548.57 m^3^ between August’s forecasted and observed values. In September the predicted flow was close to the observed value and differed by 2758.31 m^3^. The predicted time of occurrence of the flood peak differed by 1 month from the observed value and the predicted value of the flood peak differed from the observed value by 14,077.69 m^3^. In other months, the predicted value fluctuates with the observed value but remains very close. From the evaluation index, it can be found that the values predicted by Hankou station in 1998 are close to the observed values as a whole, but there is still a big gap in the flood prediction. The R2 value reaches 0.83; the LMI reaches 0.67; the WI value is low, only 0.95; and the RMSE value is 7849.91 m^3^. Furthermore, the slope of the regression line of the scatter plot is big, and therefore the overall prediction results have some degree of accuracy, but there is a big difference between the prediction and observation results of the flood peak. Figure 10C,D show the prediction results of the Hankou station in 2016 when the Yangtze River’s streamflow was smaller than that during the 1998 flood. In 2016, the peak flow occurred in July (49,355 m^3^), and the duration of the flood was shorter than that of the 1998 flood. The flood’s peak passed by August and dropped to 49,355 m^3^ in September. The model predictions are approximately the same as the observed values; only in June is there is a large deviation between the predicted value and the observed value (a difference of 13,493.98 m^3^). The same issue can also be observed in Fig. 11C. In terms of the overall trend, the forecast results for 2016 all show that the June forecast is greater than the observed value, while the July forecast is less than the observed value. Comparing the forecast results for 1998, it can be observed that both the June and July forecasts for 1998 are smaller than the observed values. This result is because the generalization of the model is considered and no overfitting can occur. From this perspective, it can be found that the model performs as expected. Additionally, the predicted values for July and August are very close to the observed values. The peak flood prediction in July has only a 909.99 m^3^ difference from the observed value. The overall predicted RMSE was 5228.97 m^3^, the R2 value reached 0.80, the LMI was 0.64, and the WI was 0.96. Thus, better forecasting results are obtained for the 2016 flood, not only in non-flood months but also in flood months, and the forecasts can be considered accurate.

The 1998 floods not only caused damage in the middle reaches of the Yangtze River, but also resulted in persistently high water levels in the lower basin compared to previous years, with monthly flows of nearly 80,000 m^3^ observed at the Datong station, which continued from July to August and remained above 60,000 m^3^ until September. Figure 11A,B shows the results of the 1998 flow forecast using the model with the same parameters as the Hankou station forecast model above. The predicted values above and below the observed values from January to June, and the predicted values for July, August, and September are close to the observed values. A large difference of 10,655.86 m^3^ is seen between July’s predicted and observed values, and the peak flow prediction for August was accurate and differed from the observed value by 3186.73 m^3^. Meanwhile, the predicted values from September to December are almost the same as the observed values. When the floods occurred the model predictions for June and July, differed from the observed values in both trend and absolute difference, but the overall flood trend was well predicted. This indicates that the model gives good predictions even in the face of a very large flood such as that of 1998 without overfitting. The regression line of the predicted and observed values is very close to the red line representing the case where predicted values perfectly match observed values.

Figure 11C,D show the 2016 prediction results of the Datong station. It can be seen that the overall predicted values are close to the observed values. This phenomenon has similarities to the predicted trends for Hankou 2016 above. Although the predicted values for the flood season differ from the observed trends, the overall predicted trends are accurate and ensure the reliability of the model. For example, June’s predicted values are larger than its observed values, while July’s predicted values are smaller than its observed values. The predicted results for the 2018 Datong station are shown in Fig. 11E,F. The observed flood peak occurred in July with a maximum flow of approximately 43,000 m^3^/s and the model predictions gave slightly higher predicted values. The values for R2, LMI and WI are excellent and the RMSE is 3489.90 m^3^. Figure 11B,D,F show that the model gives more accurate predictions at Datong Station in 1998, 2016 and 2018. Both predictions have an R2 of approximately 0.88, an LMI greater than 0.65, a WI of 0.97 and RMSE values between 3000 and 7000 m^3^, which means that the predictions are accurate.

## Comparisons and analysis

The proposed model uses different segmentation methods to divide the training set’s samples. The segmented time series data of different lengths contain input and output sequences and are fed to the neural network for training. Finally, the trained network is verified by using a validation set. Disordering the training data is a necessary operation for the neural network since the disordered data can increase the stability and robustness of the neural network and prevent the model from converging to the local optimal solution too quickly and overfitting. Due to the different lengths of the time series, the implicit links contained in the time series are also different. The streamflow data and ENSO data have corresponding implicit rules in different period scales, and these implicit rules directly affect the training effect and prediction accuracy of the model. Streamflow varies on an annual cycle. Therefore, in our experiment, we select the period multiplied by the annual cycle to observe the prediction accuracy of different cycle time series, which have four lengths: 6 months, 12 months, 18 months, and 24 months. By doing this, we can make accurate predictions with results close to those obtained by non-time series models. When the length of the selected time series is too long, the number of time series segments that can be segmented from the data decreases, and the monthly streamflow data from 1952 to 2016 is too small for machine learning. Therefore, the aforementioned lengths of time series data are selected. The Conv LSTM encoder–decoder GRU model, which is the most complex model, is selected for comparison. Below, we present the prediction results of the Conv LSTM encoder–decoder GRU model on the streamflow + ENSO dataset for the Hankou station and Datong station for 1998–2016 with different time series lengths.

**Figure 12:**
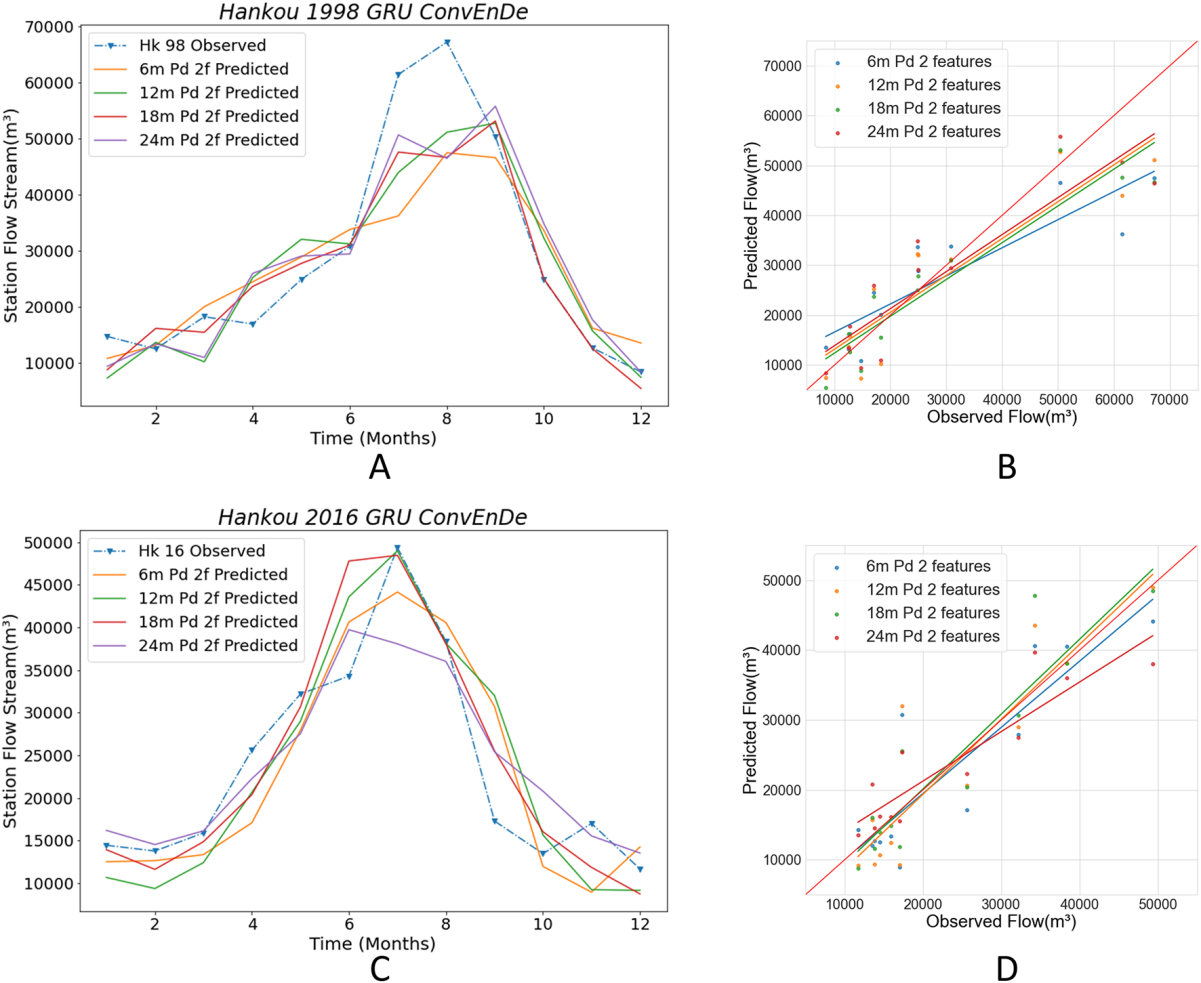
Comparison among the Conv encoder–decoder GRU with different periods in Hankou station. The solid lines in **A, C** plot the predicted values using different periods of data, and the blue dashed lines are the observed values. (**B, D**) Scatter plots of predicted and observed values for the four periods of data.

Figure 12 shows the prediction results for the four time series lengths for the Hankou station in 1998 and 2016. Figure 12A,B show the predicted streamflow of the Hankou station in 1998 using the streamflow data. It can be seen that the predicted values obtained with the four lengths are close to each other. The overall trend of the predicted values obtained using 6 m-min-pd is flat. The overall trend of predicted values using 12 m-min-pd, 18 m-min-pd, and 24 m-min-pd fluctuates widely. 24 m-min-pd is closest to the observed value in the peak flow prediction, followed by 12 m-min-pd and 18 m-min-pd. The largest differences between predicted and observed flood were obtained for 6 m pd and 18 m pd with almost identical values from September to December. In Fig. 12B, the regression lines for 12 m-min-pd, 18 m-min-pd, and 24 m-min-pd are nearly identical to the red line; meanwhile, the regression line for 6 m-min-pd is very far from the red line. By observing Table 1, it can be found that the four evaluation indexes of 18 m-min-pd are better than the results obtained from other datasets. The RMSE reaches 7849.91 m^3^, the WI value reaches 0.95, the LMI value is significantly different from those of other cases, and the LMI is 16–24% higher than in other cases, and the R2 is 4–15% higher than those of other cases. The best prediction was achieved in the 1998 results for 18 m-min-pd at Hankow Station. Figure 12C,D show the predicted streamflow of the Hankou station in 2016 using the streamflow data. It can be seen that the most accurate prediction is 12 m-min-pd, followed by 18 m-min-pd, 6 m-min-pd, and finally 24 m-min-pd. Similarly, the scatterplots and corresponding regression curves in D show that the results for 6 m pd, 12 m pd, and 18 m pd are very close to the red line; meanwhile, 24 m-min-pd has the poorest results and deviates greatly from the red line. Furthermore, 24 m-min-pd has the smallest RMSE value (5196.74 m^3^), with 18 m-min-pd close behind; the other two cases have large RMSEs. Additionally, 18 m-min-pd has the highest WI and LMI values (0.96 and 0.64, respectively). Therefore, 18 m-min-pd showed the best results and highest accuracy for predictions. It can be seen that contact ENSO and streamflow data have features that are easy for machine learning models to grasp on the 18-min-pd time scale. On the 6-min-pd dataset, however, the model performs the worst, especially in the 1998 prediction results. Because the field of view is too small in the model training, it is not possible to observe the complete cycle variation at one time, which has an important impact on the formation of the correct fitting parameters. Thus, this leads to worse prediction results in 1998 when the extreme values deviate more severely.

**Table 1:** Comparison of criteria in Conv encoder–decoder GRU with different periods in Hankou station.

Year		6 m min pd	12 m min pd	18 m min pd	24 m min pd
1998	RMSE	10,224.25	8536.23	7849.91	8568.73
	WI	0.89	0.94	0.95	0.94
	LMI	0.54	0.58	0.67	0.58
	R2	0.72	0.80	0.83	0.80
2016	RMSE	5974.99	6157.50	5228.97	5196.74
	WI	0.94	0.94	0.96	0.94
	LMI	0.53	0.54	0.64	0.61
	R2	0.74	0.72	0.80	0.80

**Figure 13:**
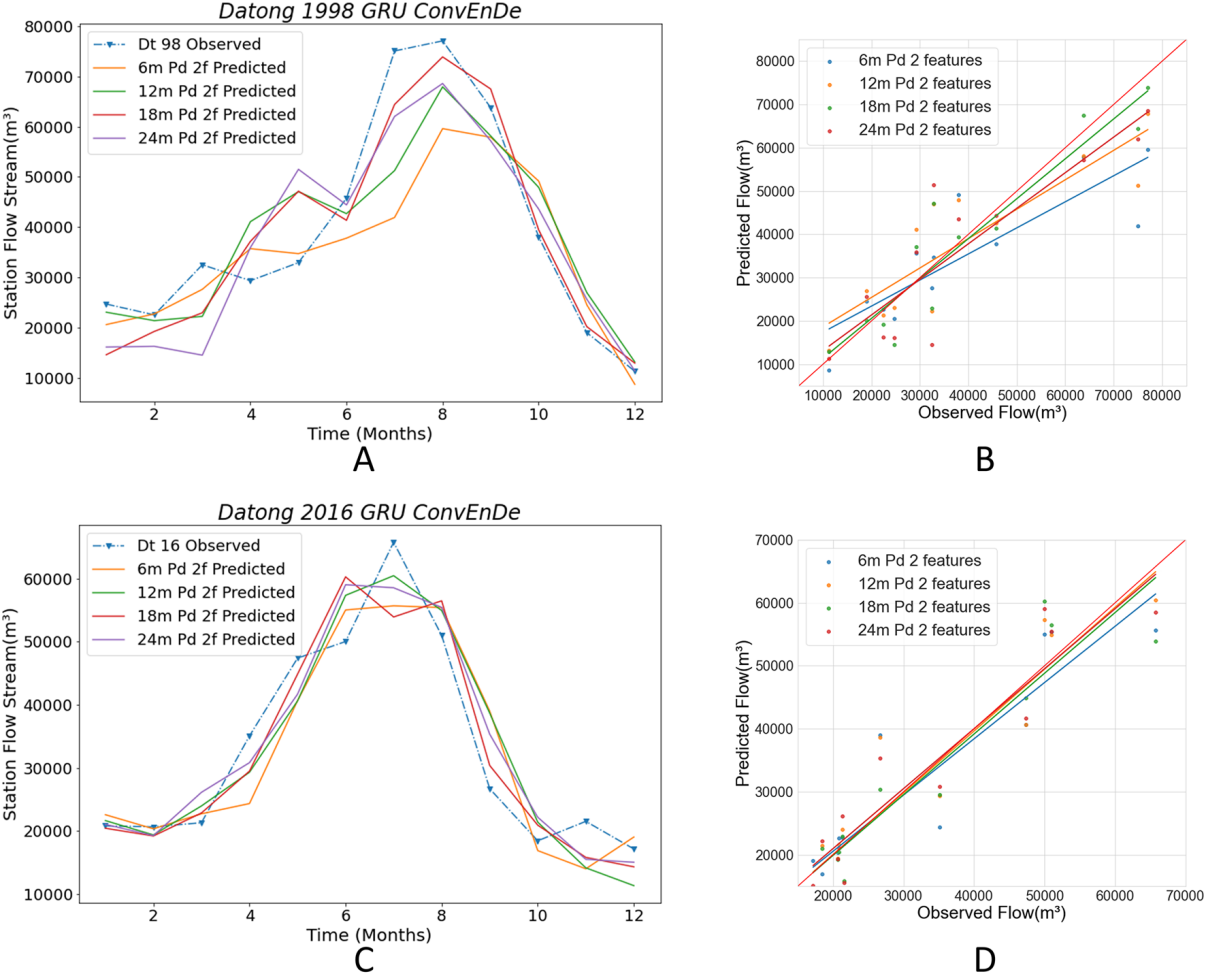
Comparison among the Conv encoder–decoder GRU with different periods in Datong station. The solid lines in **A, C** plot the predicted values using different periods of data, and the blue dashed lines are the observed values. (**B, D**) Scatter plots of predicted and observed values for the four periods of data.

Figure 13 shows the prediction for the four segmentation methods of streamflow + ENSO data at the Datong station. Table 2 also shows that the 18 m-min-pd indicator is excellent, with a WI of 0.97 and an LMI of 0.66. The maximum WI is 0.94 and the maximum LMI is 0.52. Figure 13C,D show the results of the four data sets for predicting 2016 flows at the Datong station, which are very close, with poor overall prediction accuracy for 6 m-min-pd. 12 m-min-pd, 18 m-min-pd, and 24 m-min-pd predictions are similar. The 18 m-min-pd and 24 m-min-pd predicted peak flows 1 month earlier than observation. 12 m-min-pd was accurate for the month of peak flow, and followed observed values; meanwhile, 18 m-min-pd has the largest difference between forecasted and true values in July. In Fig. 13D, the regression line for 6 m-min-pd is slightly off the red line, while the regression lines for the remaining results are very close to the red line. The evaluation indicators presented in Table 2 show that all four results have reached very high values, with the best results for 18 m-min-pd and 24 m-min-pd.

**Table 2:** Comparison of criteria in Conv encoder–decoder GRU with different period in Datong station.

Year		6 m min pd	12 m min pd	18 m min pd	24 m min pd
1998	RMSE	12,074.85	10,430.62	7249.87	9954.34
	WI	0.88	0.92	0.97	0.94
	LMI	0.52	0.52	0.66	0.52
	R2	0.67	0.75	0.88	0.77
2016	RMSE	6618.38	5957.79	5610.82	5462.29
	WI	0.95	0.97	0.97	0.97
	LMI	0.62	0.63	0.68	0.66
	R2	0.82	0.86	0.87	0.88

Combining the prediction results for 2 years, we can find that 18 m-min-pd outperforms the other datasets in most cases, and gives predictions suitable for later reference. We can conclude that the model with 18 m-min-pd performs well on the streamflow + ENSO dataset.

**Figure 14:**
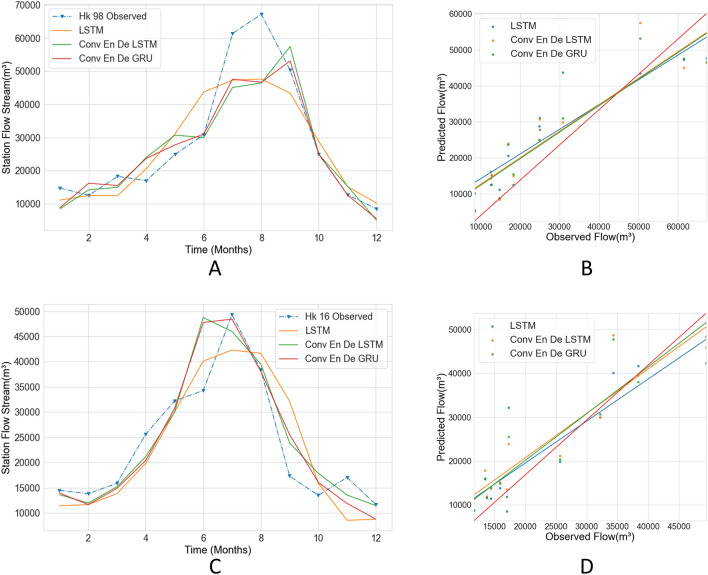
Comparison with different models in Hankou station. The solid lines in **A, C** plot the predicted values using different models, and the blue dashed lines are the observed values. (**B, D**) Scatter plots of predicted and observed values for three models.

**Figure 15:**
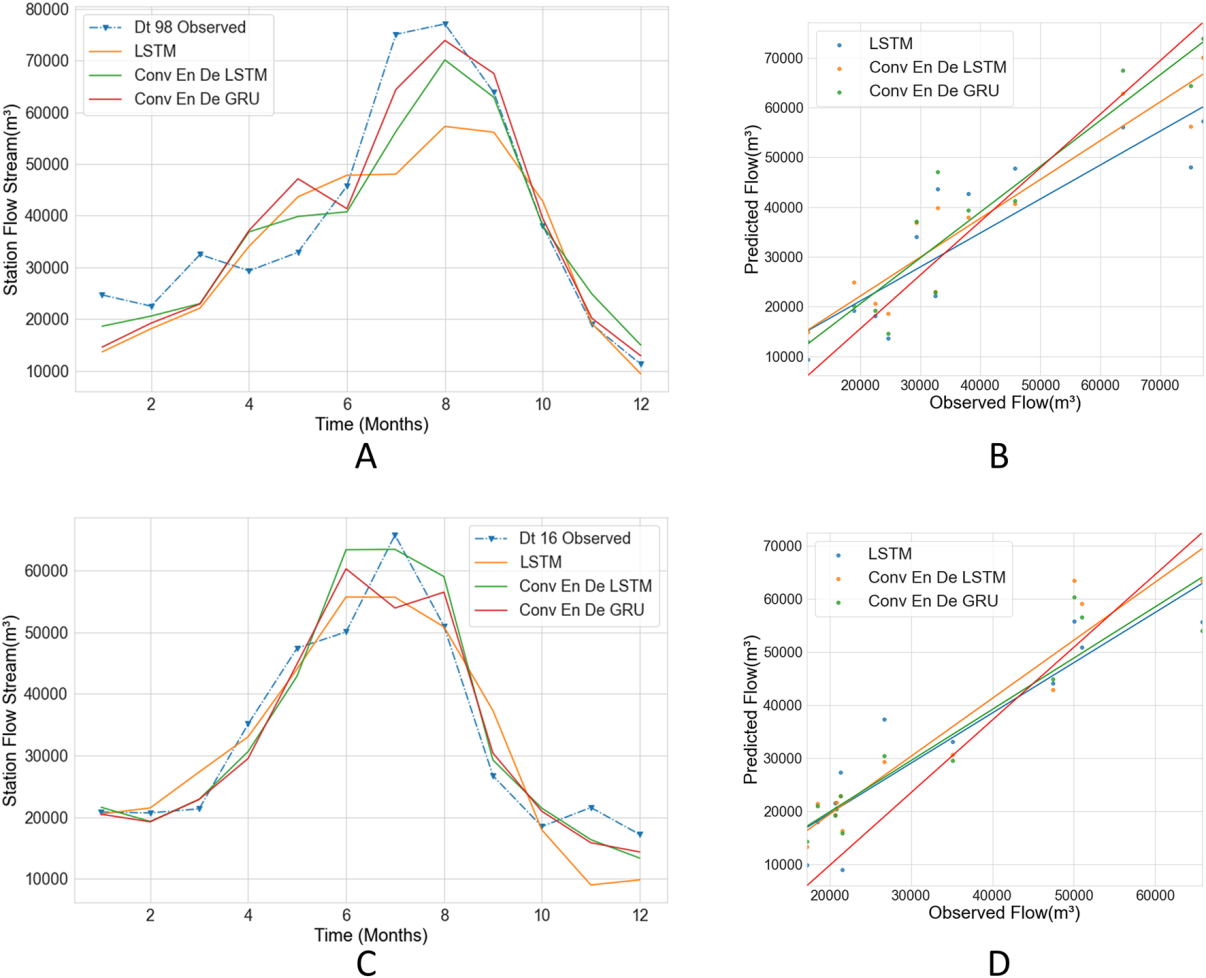
Comparison with different models in Datong station. The solid lines in **A, C** plot the predicted values using different models, and the blue dashed lines are the observed values. (**B, D**) Scatter plots of predicted and observed values for three models.

In this study, we experiment with stacked LSTM, Conv LSTM encoder–decoder LSTM, Conv LSTM encoder–decoder GRU and select the model with the most accurate predictions. The results are presented in Figs. 14 and 15 and Tables 3 and 4. Figure 14A shows the line graphs of the predictions of the three models for the Hankou station, and it can be seen that the predictions of LSTM are quite different from those of the other models. Conv LSTM encoder–decoder LSTM and Conv LSTM encoder–decoder GRU have similar predictions, but with Conv LSTM encoder–decoder LSTM having better predictions in September, and Conv LSTM encoder–decoder GRU having better predictions in July. Meanwhile, the regression lines of the three models are similar, and only the LSTM value is slightly far away from the red line, which means that the overall performance of the three models is similar. The R2 values of the three models are around 0.8, which means that the predictions of the three models are close to the observed values; additionally, the Conv LSTM encoder–decoder GRU model has the best results regarding the other three evaluation indexes. Figure 14C shows the predictions of the three models on the monthly streamflow of the Hankou station in 2016. The predictions of the three models are similar from January to May. Stacked LSTM’s prediction during the flood season is different from those of the other models and is far from the observed value; meanwhile, the LSTM prediction differs from the observed value by 7038.91 m^3^, the Conv LSTM encoder–decoder LSTM prediction is similar to the observed value, and the Conv LSTM encoder–decoder LSTM prediction is different from the observed value by 0.67 m^3^. The flood peak occurs in June, and the observed flood peak occurs in July. Conv LSTM encoder–decoder GRU’s flood peak prediction is the same as the observed flood peak. Figure 14D shows that the LSTM model has a different regression line than the other models. Table 3 shows that Stacked LSTM has the poorest results and the worst fit to observations; the other models perform better and the predictions are nearly identical. Combining the 2-year prediction data from the Hankow station, it can be concluded that the prediction of Conv LSTM encoder–decoder GRU is slightly better than that of Conv LSTM encoder–decoder LSTM, and the prediction of Stacked LSTM is worst.

**Table 3:** Comparison of criteria with different models in Hankou station.

Year	Model	Conv En De GRU	Conv En De LSTM	Stacked LSTM
**Hankou**				
1998	RMSE	7849.91	8675.98	8757.40
	WI	0.95	0.94	0.93
	LMI	0.67	0.61	0.58
	R2	0.83	0.80	0.79
2016	RMSE	5228.97	5216.75	6184.42
	WI	0.96	0.95	0.93
	LMI	0.64	0.65	0.51
	R2	0.80	0.80	0.72

**Table 4:** Comparison of criteria with different models in Datong station.

Year	Model	Conv En De GRU	Conv En De LSTM	Stacked LSTM
**Datong**				
1998	RMSE	7249.87	7697.48	11,542.46
	WI	0.97	0.96	0.90
	LMI	0.66	0.65	0.50
	R2	0.88	0.86	0.69
2016	RMSE	5610.82	5400.94	6523.28
	WI	0.97	0.97	0.96
	LMI	0.68	0.70	0.65
	R2	0.87	0.88	0.83

Figure 15 shows a comparison of the predictions of the three models for 1998 and 2016 for the Datong station. The non-flood season shows similar results for the three models. The flood season shows that the Conv LSTM encoder–decoder GRU prediction is closest to the observed value, followed by the Conv LSTM encoder–decoder LSTM. The worst is prediction is produced by Stacked LSTM, and the slope of the regression line for Conv LSTM encoder–decoder GRU on scatter plot Fig. 15B is closest to the red line, followed by Conv LSTM encoder–decoder LSTM. Stacked LSTM has the largest difference. In Fig. 15C, it is found that the results of the three models are still similar during the non-flood season, with the Conv LSTM encoder–decoder LSTM results being closer to the peak flow during the flood season; Stacked LSTM’s predicted streamflow has a larger gap between the peak flow and the flood. Conv encoder–decoder GRU's flood predictions fall between the results of the other models, with the predicted flood peak occurring in June, 1 month away from the observed flood peak, with a small difference in flood flows, but the largest difference between the predicted July flows and the observed values. The regression line for the Conv LSTM encoder–decoder LSTM is closest to the red line in Fig. 15D. The four evaluation indicators listed in Table 4 show that the 1998 forecast results from Conv LSTM encoder–decoder GRU are the best, and those from Conv LSTM encoder–decoder LSTM are the best for the 2016 forecast.

By comparing the model predictions, Conv LSTM encoder–decoder GRU has the best 2-year prediction among the three models. Conv LSTM encoder–decoder GRU has a similar prediction to Conv LSTM encoder–decoder LSTM with a 1-year prediction, Conv LSTM encoder–decoder LSTM has the best prediction with a 1-year prediction, and Stacked LSTM has the worst prediction in all cases. Conv LSTM encoder–decoder GRU performs slightly better than Conv LSTM encoder-decoder LSTM. Because of the addition of using ConvLSTM as the encoder structure in the stacked LSTM, the feature extraction ability of the model is enhanced. Because the difference between GRU and LSTM is smaller, the difference between the models trained as the decoder is smaller. The main factor to enhance the prediction accuracy is to improve the model using ConvLSTM as the encoder. This is consistent with our expectation of improving the prediction accuracy of the model by enhancing the model to extract data features.

**Figure 16:**
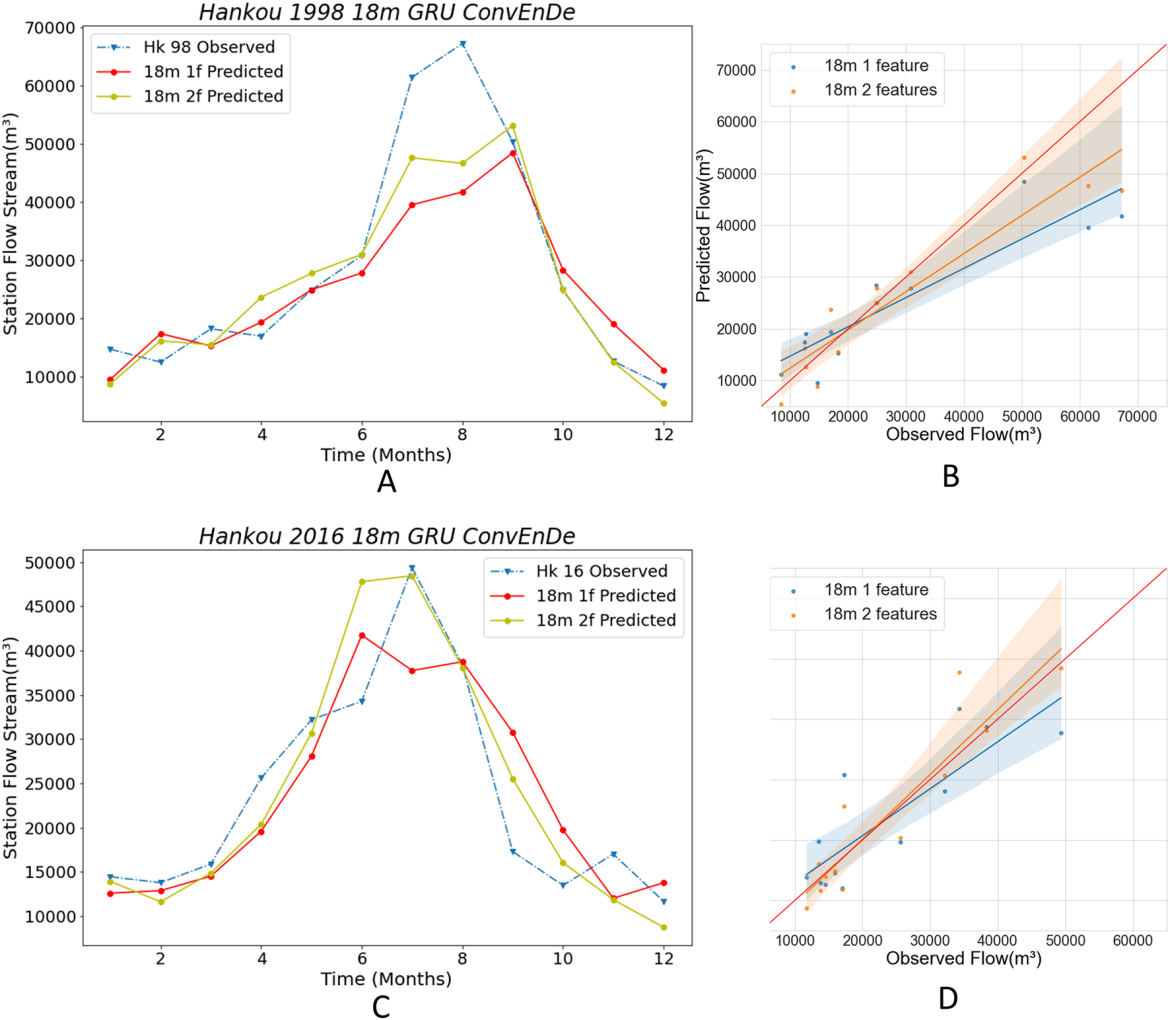
Comparison with different features in Hankou station.The solid lines in **A, C** plot the predicted values using different numbers of feature data, and the blue dashed lines are the observed values. (**B, D**) The scatter plots corresponding to **A, C**.

**Figure 17:**
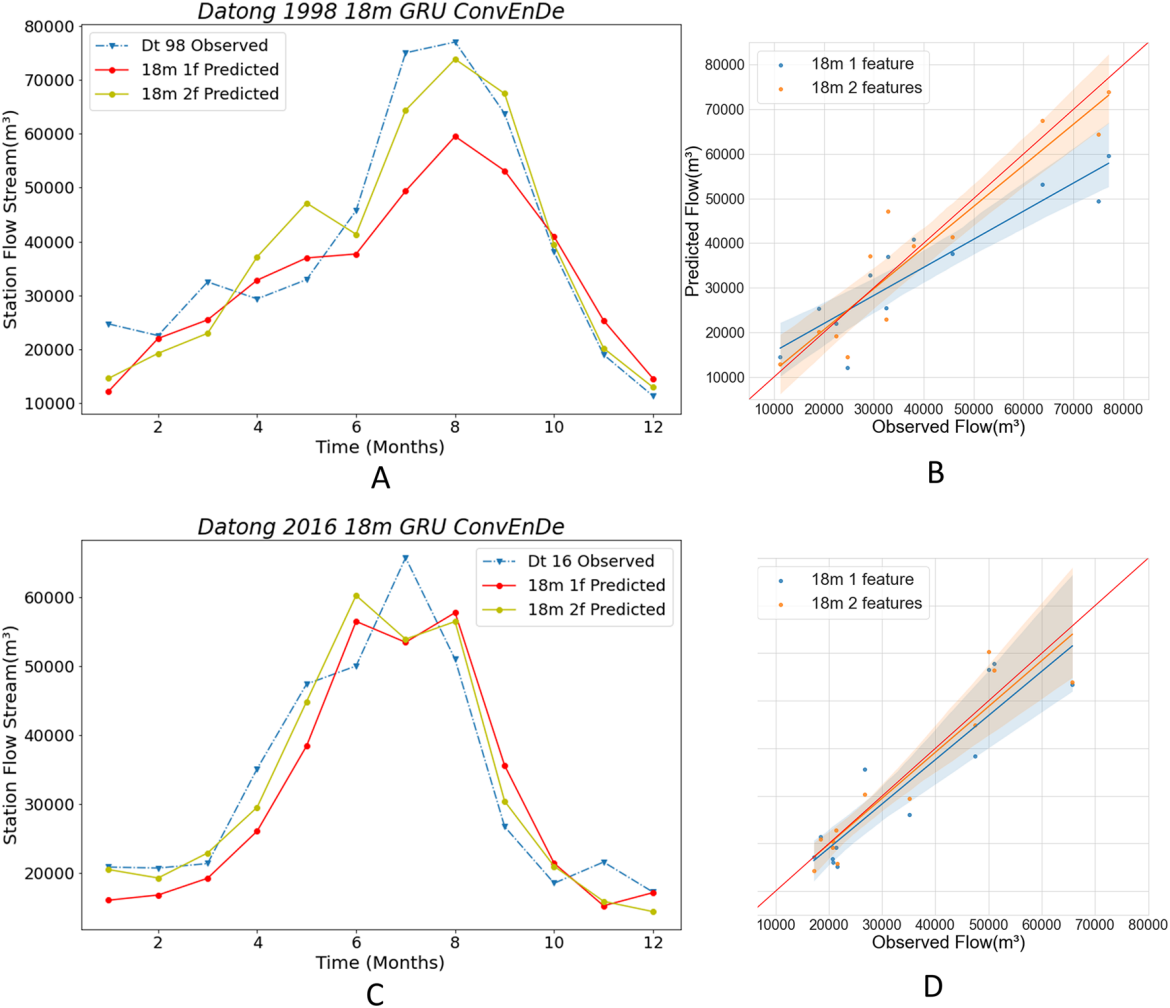
Comparison with different models in Datong station. The solid lines in **A, C** plot the predicted values using different numbers of feature data, and the blue dashed lines are the observed values. (**B, D**) The scatter plots corresponding to **A, C**.

In this paper, we introduce ENSO values that are implicitly related to the streamflow data, in addition to the previous machine learning approach of using only streamflow data for training and prediction. Through this, we can enhance the training effect by increasing the data dimensions and get more accurate monthly streamflow predictions, and hopefully more accurate flood predictions. The best-performing Conv LSTM encoder–decoder GRU model is used in this next experiment, and the best-performing 18 m-min-pd data partitioning method is used to compare the difference in prediction results between the ENSO + streamflow dataset and the streamflow dataset. The experimental results are presented in Figs. 16 and 17 and Tables 5 and 6, where A and C plot the line graphs of monthly flows, and B and D present scatter plots, regression lines, and confidence intervals.

**Table 5:** Comparison of criteria with different features in Hankou station.

Year		1 feature	2 features
**Hankou**			
1998	RMSE	10,272.88	7849.91
	WI	0.89	0.95
	LMI	0.58	0.67
	R2	0.71	0.83
2016	RMSE	6453.44	5228.97
	WI	0.91	0.96
	LMI	0.51	0.64
	R2	0.70	0.80

**Table 6:** Comparison of criteria with different features in Datong station.

Year	Model	1 feature	2 features
**Datong**			
1998	RMSE	10,951.82	7249.87
	WI	0.90	0.97
	LMI	0.51	0.66
	R2	0.72	0.88
2016	RMSE	6905.13	5610.82
	WI	0.95	0.97
	LMI	0.57	0.68
	R2	0.81	0.87

Figure 16A plots the 1998 prediction results from Hankou station. It can be seen that the results from the 2features data are closer to the observed values during the flood months, while the October–November prediction is almost equal to the observed values. From Fig. 16B, we also find that the regression line of the 2features results is closer to the red line, but the confidence intervals are similar in both cases. By comparing the four evaluation indicators of Table 5, it can be found that the RMSE of 2features is reduced by 2422.97 m^3^, WI is increased by 6% to 0.95, LMI is increased by 16% to 0.67, and R2 is increased by 17% to 0.83. In Fig. 16C, the prediction results of Hankow station in 2016 are presented. The model using 2features data accurately predicts the timing and streamflow of flood peaks, while the 1features model flood peak prediction differs by 1 month from the observed value and gives a value that differs significantly from the observed value in the month in which the flood peak occurs. Besides, in non-flood seasons, the 2features predictions are closer to the observations. In Fig. 16D it can be seen that the confidence intervals are about the same, but the regression line for 2features is very close to the red line; meanwhile, the regression line for 1features differs more from the red line. Moreover, all the four evaluation indices are greatly improved (Table 5): the RMSE is reduced by 1224.47 m^3^, WI is increased by 5% to 0.96, LMI is increased by 25% to 0.64, and R2 is increased by 14% to 0.80. Based on these results, we can obtain the monthly streamflow rate of the model in Hankou in 1998 after adding ENSO data. Predictions have significantly improved, with better results not only in non-flood months but also in flood months.

Figure 17A plots the 1998 prediction results of the Datong station. The 2features data make the prediction much more accurate than 1 feature data, and the predictions for July, August, and September are very close to the observations; meanwhile, the 1features data make the predictions significantly different from the observations. In October–December, the prediction of 2features is almost equal to the observed value. Figure 17B shows that the regression line for the 2features is very close to the red line. On Table 6, it is clear that the 2features evaluation index has improved significantly, with the RMSE shrinking by 3701.95 m^3^, WI increasing by 8% to 0.97, LMI increasing by 29% to 0.66, and R2 increasing by 22% to 0.88. In Fig. 17C, it is clear that the two eigenvalues yield similar prediction results. The 2features result flood peak occurs in June, the 1features result flood peak occurs in August, and the observed flood peak occurs in July; meanwhile, the two predictions for July are almost identical and differ significantly from the observations. The comparison of the predicted flood peaks shows that the 2features results are closer to the observed maximum flows. In non-flood months, most of the 2features results are more accurate. The regression lines for the results on Fig. 17D for both data are very close, but the regression line for the predicted results for 2features is slightly more accurate. A slight improvement in the evaluation metrics for 2features over 1feature can also be seen through Table 6. Ultimately, these results illustrate that the accuracy of the prediction is improved by adding ENSO data, with a significant improvement in the 1998 prediction and a small improvement in the 2016 prediction.

In the above comparison, the addition of ENSO data to the 18 m-min-pd division in the Conv LSTM encoder–decoder GRU significantly improves the prediction accuracy.

Futhermore, to investigate the effect of Enso values on flow prediction, the predicted monthly flows for both Hankow and Datong stations in 1998 and 2016 are compared under three models and four data divisions, These results are shown in Tables S1–4. Tables S1 and S2 show the comparison of the four-evaluation metrics for the prediction results, and Tables S3 and S4 show the comparison of the maximum flows for the prediction results. Tables S1, S2 show that the prediction results are all improved to varying degrees by the addition of ENSO values, and when the model originally had poor predictions, adding data feature values will result in a greater improvement in the accuracy of the predictions, such as stacked LSTM for Hankou station in 2016 using 6 m-min-pd data and Datong station in 1998 and 2016. When the model's original predictions are more accurate, the effect of increasing the value of the data features on the prediction accuracy is diminished, as observed in the prediction results of Conv encoder–decoder LSTM and Conv encoder–decoder GRU. Besides, comparing the 1998 prediction results with the 2016 prediction results, the prediction error in 1998 is found to be larger than the prediction error in 2016 on both hydrological stations. This is found on the two datasets. The differences that appear on WI, LMI, and R2 are small, ranging from 2 to 10% on average. Meanwhile, the differences in the RMSE values are larger. In Datong, the mean value of RMSE in 1998 is 4334.98 m^3^ larger than the mean value of RMSE in 2016. This difference is 3850.99 m^3^ in Hankou. It is evident that the difference in the overall trend of the model in predicting mega-floods and large floods is small. By observing Tables S3, and S4 it can be found that the difference in RMSE originates from the extreme anomalies during the flood. In the case of the maximum flow prediction, the maximum flows predicted by each model were closer to the observed maximum flows with the addition of ENSO data, and the months in which the maximum flows were predicted to occur were more accurate. However, even after the introduction of ENSO, the model still has some gaps in the prediction of extreme outliers. Then better prediction of extreme outliers while ensuring the model's overfitting is the direction of future research.

**Figure 18:**
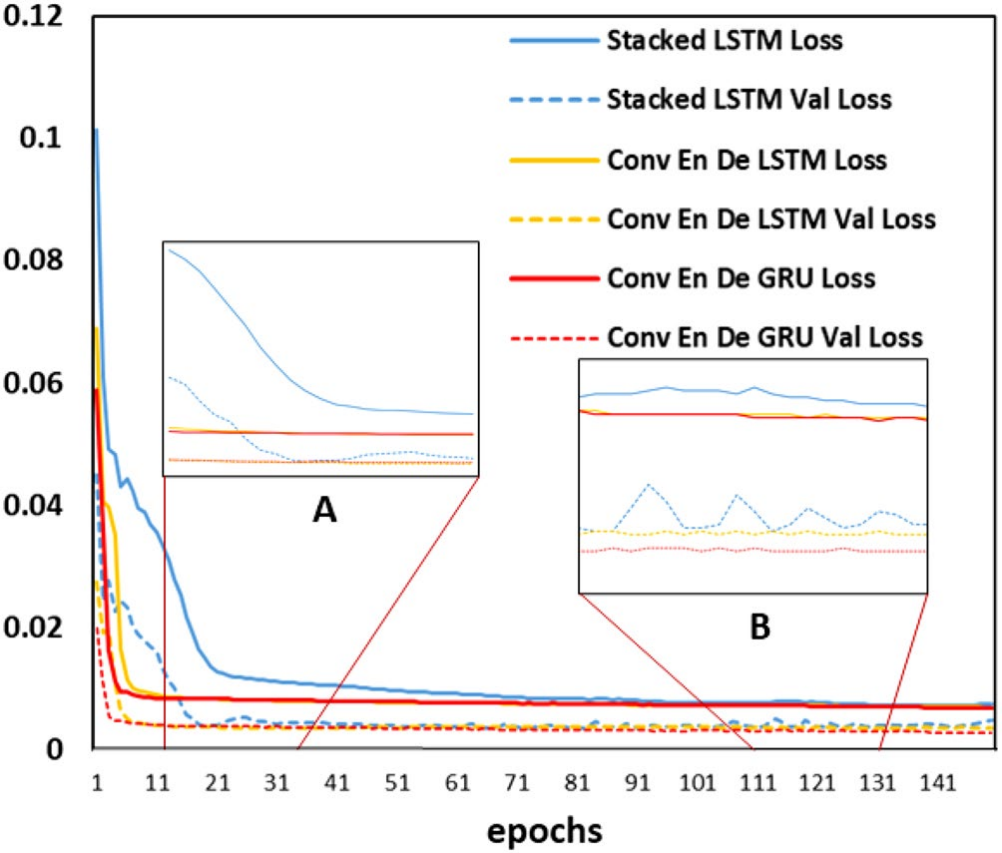
Comparison of model loss changes.

Figure 18 shows the variation of loss and valid loss of the three models during the training process. Observing loss and valid loss is a way to understand the training process of a model in machine learning. It is a good indication of how well the models were trained and whether overfitting. Occurred during training than observing the model prediction results. The ANN in this has the same parameter settings for this assessment. The solid line in the figure is the Loss and the dashed line is the Val Loss. Figure 18A finds that the Conv LSTM encoder–decoder LSTM and Conv LSTM encoder–decoder GRU models have similar declines in loss and valid loss, and both are faster than in the stacked LSTM. Figure 18B shows that the trend of the parameters tends to smooth out and the models are trained at their best. It can be found that the Conv LSTM encoder–decoder model has a very small variation in loss and valid Loss, which indicates that the model fits better and also gives more stable predictions. In contrast, the values of loss and valid loss of the stacked LSTM fluctuate significantly, indicating that the model is overfitted to a certain extent, and the predictions given by the model will vary greatly with the number of training sessions. It can also be seen that the Conv encoder–decoder GRU has the lowest valid loss, this also shows that the model has the best performance.

## Conclusion

In this paper, three network structures, stacked LSTM, Conv LSTM encoder–decoder LSTM, and Conv LSTM encoder–decoder GRU, are trained using two eigenvalues datasets, ENSO and monthly streamflow, to predict the monthly streamflow of the Yangtze River in Hankou and Datong stations in 1998, 2016 and 2018. The best results were obtained with the Conv LSTM encoder–decoder GRU: the R2 exceeded 0.80, the RMSE was less than 8000 m^3^, the WI was over 0.95, and the LMI over 0.65, indicating more accurate flood prediction.

This paper compares the prediction results of the three network structures for two flood years, 1998 and 2016, and shows that the prediction accuracy of all three network structures is improved by adding ENSO data. The improvement of Conv LSTM encoder–decoder LSTM and Conv LSTM encoder–decoder GRU is smaller than that of stacked LSTM. The final prediction results achieved a maximum evaluation index of R2 = 0.88, LMI = 0.66, and WI = 0.97 and a minimum prediction difference of 389 m^3^ for the flood peak. We performed statistical calculations on the parameters in Tables S1 and S2 and obtained an overall improvement of 21.91% in the evaluation metrics for the stacked LSTM model after the introduction of the ENSO value, 10.87% for the Conv encoder–decoder LSTM model, and 10.87% for the Conv encoder–decoder The overall evaluation metrics of the GRU model improved by 11.91%. It can be seen that the enhancement of the dataset results in different magnitudes of improvement in the prediction for each of the three network structures; this is because Conv LSTM encoder–decoder LSTM and Conv LSTM encoder–decoder GRU already have strong feature extraction capabilities for time series, while Stacked LSTM has relatively weak feature extraction capabilities. The added deep connection between ENSO data and flow data enables the network structure to extract more information, thus compensating for the time series feature extraction deficiency to some extent and greatly improving the accuracy of prediction. We experimented with a deep learning model using Conv LSTM as Encoder to predict flood data. The results show that the prediction results are improved by using the Conv LSTM encoder–decoder model. Not only is the accuracy of the prediction results improved, but the degree of model fit is increased. The overfitting of the model was reduced.

We found that the neural network model predicts the middle and lower reaches of the Yangtze River represented by the flows at Hankou and Datong stations. By adding ENSO data to the streamflow data, the prediction ability of each model on different parameters is greatly improved, which reveals that there is an implicit relationship between ENSO and flow data that can be learned by the neural network. Comparing the prediction results for 1998, 2016 and 2018, it is found that the error for 1998 is larger than that for 2016 and 2018. Furthermore, the predicted results for 1998 are all smaller than the observed values, this does not occur for the other years. The reason for this discrepancy may be that the flow in the Yangtze River basin is increasingly influenced by human factors over time, such as the Three Gorges Dam. Perhaps these factors are already implicit in the flow data, allowing the model to still give good results. However, this makes the model's predicted value in recent years will be greater than the observed value. The prediction accuracy might be improved if relevant data about human activities are added. The difference between the streamflow of the Yangtze River in the last century and the streamflow changes in the current century is due to this influence, which leads to the fact that the prediction model cannot learn similar unnatural river streamflow changes simply by adding ENSO data. We note that the number of streamflow data samples collected is only about 700, which is small for machine learning. Augmenting the model with ENSO data can be seen as augmenting the training set and compensating for this lack of data.

The variation in streamflow volume in the Yangtze River is not only related to the ENSO data but also many other variables; thus, the data can be enhanced by adding more variables, which would make the prediction more accurate. Different regions in the Yangtze River basin have different relationships with climate change, and different locations in the Yangtze River have different relationships with upstream streamflow; thus, more sites could be used for joint prediction. It is also possible to try using the improved model in combination with methods such as numerical analysis, to obtain better predictions.

## Supplementary information

Supplementary Informations.
