# Supplementary material for: Prediction of Yangtze River streamflow based on deep learning neural network with El Niño–Southern Oscillation
Source: Sci Rep. 2021 Jun 3;11:11738. doi: 10.1038/s41598-021-90964-3 (PMC8175427; doi:10.1038/s41598-021-90964-3)
Supplement: Supplementary file 1 — Supplementary Informations. [file 41598_2021_90964_MOESM1_ESM.docx]

Table 1 The criteria of Hankou station

|  |  |  |  |  |  |  |  |  |  |  |  | | | | | | | | | | | | | | | |
| --- | --- | --- | --- | --- | --- | --- | --- | --- | --- | --- | --- | --- | --- | --- | --- | --- | --- | --- | --- | --- | --- | --- | --- | --- | --- | --- |
| **Hankou Station** | | | | | | | | | | |  | | | | | | | | | | | | | | | |
|  |  |  | **6m min pd** | | **12m min pd** | | **18m min pd** | | **24m min pd** | |  | | | | | | | | | | | | | | | |
| **Model** | **Year** |  | **1 feature** | **2 features** | **1 feature** | **2 features** | **1 feature** | **2 features** | **1 feature** | **2 features** |  | | | | | | | | | | | | | | | |
| **Conv encoder– decoder GRU** | **1998** | **RMSE** | 11885.94 | 10224.25 | 11069.12 | 8536.23 | 10272.88 | 7849.91 | 10528.94 | 8568.73 |  | | | | | | | | | | | | | | | |
|  |  | **WI** | 0.84 | 0.89 | 0.87 | 0.94 | 0.89 | 0.95 | 0.89 | 0.94 |  | | | | | | | | | | | | | | | |
|  |  | **LMI** | 0.54 | 0.54 | 0.55 | 0.58 | 0.58 | 0.67 | 0.54 | 0.58 |  | | | | | | | | | | | | | | | |
|  |  | **R2** | 0.62 | 0.72 | 0.67 | 0.80 | 0.71 | 0.83 | 0.70 | 0.80 |  | | | | | | | | | | | | | | | |
|  | **2016** | **RMSE** | 6840.26 | 5974.99 | 6449.84 | 6157.50 | 6453.44 | 5228.97 | 6227.46 | 5196.74 |  | | | | | | | | | | | | | | | |
|  |  | **WI** | 0.90 | 0.94 | 0.91 | 0.94 | 0.91 | 0.96 | 0.93 | 0.94 |  | | | | | | | | | | | | | | | |
|  |  | **LMI** | 0.52 | 0.53 | 0.53 | 0.54 | 0.51 | 0.64 | 0.53 | 0.61 |  | | | | | | | | | | | | | | | |
|  |  | **R2** | 0.66 | 0.74 | 0.70 | 0.72 | 0.70 | 0.80 | 0.72 | 0.80 |  | | | | | | | | | | | | | | | |
| **Conv encoder– decoder LSTM** | **1998** | **RMSE** | 11873.32 | 10465.17 | 10912.88 | 10003.99 | 10115.54 | 8675.98 | 9645.38 | 8623.09 |  | | | | | | | | | | | | | | | |
|  |  | **WI** | 0.84 | 0.88 | 0.87 | 0.90 | 0.89 | 0.94 | 0.91 | 0.93 |  | | | | | | | | | | | | | | | |
|  |  | **LMI** | 0.54 | 0.56 | 0.56 | 0.59 | 0.57 | 0.61 | 0.59 | 0.62 |  | | | | | | | | | | | | | | | |
|  |  | **R2** | 0.62 | 0.70 | 0.68 | 0.73 | 0.72 | 0.80 | 0.75 | 0.80 |  | | | | | | | | | | | | | | | |
|  | **2016** | **RMSE** | 6722.49 | 5347.60 | 6448.34 | 5859.16 | 6131.52 | 5216.75 | 6211.28 | 5484.72 |  | | | | | | | | | | | | | | | |
|  |  | **WI** | 0.90 | 0.95 | 0.92 | 0.94 | 0.92 | 0.95 | 0.93 | 0.94 |  | | | | | | | | | | | | | | | |
|  |  | **LMI** | 0.52 | 0.59 | 0.53 | 0.57 | 0.54 | 0.65 | 0.50 | 0.55 |  | | | | | | | | | | | | | | | |
|  |  | **R2** | 0.67 | 0.79 | 0.70 | 0.75 | 0.72 | 0.80 | 0.72 | 0.78 |  | | | | | | | | | | | | | | | |
| **Stacked LSTM** | **1998** | **RMSE** | 11343.89 | 9967.40 | 11541.24 | 9417.74 | 11820.25 | 8757.40 | 11128.30 | 9293.75 |  | | | | | | | | | | | | | | | |
|  |  | **WI** | 0.86 | 0.90 | 0.85 | 0.91 | 0.85 | 0.93 | 0.87 | 0.92 |  | | | | | | | | | | | | | | | |
|  |  | **LMI** | 0.52 | 0.54 | 0.52 | 0.57 | 0.54 | 0.58 | 0.53 | 0.53 |  | | | | | | | | | | | | | | | |
|  |  | **R2** | 0.65 | 0.73 | 0.64 | 0.76 | 0.62 | 0.79 | 0.66 | 0.77 |  | | | | | | | | | | | | | | | |
|  | **2016** | **RMSE** | 8354.20 | 5428.27 | 7404.95 | 5720.43 | 7745.91 | 6184.42 | 6997.11 | 6311.20 |  | | | | | | | | | | | | | | | |
|  |  | **WI** | 0.83 | 0.94 | 0.87 | 0.94 | 0.85 | 0.93 | 0.89 | 0.93 |  | | | | | | | | | | | | | | | |
|  |  | **LMI** | 0.38 | 0.62 | 0.51 | 0.60 | 0.38 | 0.51 | 0.44 | 0.51 |  | | | | | | | | | | | | | | | |
|  |  | **R2** | 0.49 | 0.78 | 0.60 | 0.76 | 0.56 | 0.72 | 0.64 | 0.71 |  | | | | | | | | | | | | | | | |
|  |  | | | | | | | | | | | | | | | | | | | | | | | | | |
|  |  |  |  |  |  |  |  |  |  |  |  |  |  |  |  |  |  |  |  |  |  |  |  |  |  |  |

Table 2 The criteria of Datong station

|  |  |  |  |  |  |  |  |  |  |  |  |  |  |  |  |  |  |  |  |  |  |  |  |  |  |  |  |
| --- | --- | --- | --- | --- | --- | --- | --- | --- | --- | --- | --- | --- | --- | --- | --- | --- | --- | --- | --- | --- | --- | --- | --- | --- | --- | --- | --- |
| **Datong Station** | | | | | | | | | | |  |  | | | | | | | | | | | | | | | |
|  |  |  | **6m min pd** | | **12m min pd** | | **18m min pd** | | **24m min pd** | |  |  | | | | | | | | | | | | | | | |
| **Model** | **Year** |  | **1 feature** | **2 features** | **1 feature** | **2 features** | **1 feature** | **2 features** | **1 feature** | **2 features** |  |  | | | | | | | | | | | | | | | |
| **Conv encoder– decoder GRU** | **1998** | **RMSE** | 14132.73 | 12074.85 | 12393.00 | 10430.62 | 10951.82 | 7249.87 | 10869.14 | 9954.34 |  |  | | | | | | | | | | | | | | | |
|  |  | **WI** | 0.82 | 0.88 | 0.86 | 0.92 | 0.90 | 0.97 | 0.91 | 0.94 |  |  |  |  |  |  |  |  |  |  |  |  |  |  |  |  |  |
|  |  | **LMI** | 0.43 | 0.52 | 0.46 | 0.52 | 0.51 | 0.66 | 0.50 | 0.52 |  |  |  |  |  |  |  |  |  |  |  |  |  |  |  |  |  |
|  |  | **R2** | 0.54 | 0.67 | 0.65 | 0.75 | 0.72 | 0.88 | 0.73 | 0.77 |  |  |  |  |  |  |  |  |  |  |  |  |  |  |  |  |  |
|  | **2016** | **RMSE** | 8744.39 | 6618.38 | 6902.22 | 5957.79 | 6905.13 | 5610.82 | 6730.49 | 5462.29 |  |  |  |  |  |  |  |  |  |  |  |  |  |  |  |  |  |
|  |  | **WI** | 0.91 | 0.95 | 0.95 | 0.97 | 0.95 | 0.97 | 0.96 | 0.97 |  |  |  |  |  |  |  |  |  |  |  |  |  |  |  |  |  |
|  |  | **LMI** | 0.54 | 0.62 | 0.63 | 0.63 | 0.57 | 0.68 | 0.59 | 0.66 |  |  |  |  |  |  |  |  |  |  |  |  |  |  |  |  |  |
|  |  | **R2** | 0.69 | 0.82 | 0.81 | 0.86 | 0.81 | 0.87 | 0.82 | 0.88 |  |  |  |  |  |  |  |  |  |  |  |  |  |  |  |  |  |
| **Conv encoder– decoder LSTM** | **1998** | **RMSE** | 13821.62 | 11995.99 | 11995.13 | 10219.74 | 10681.81 | 7697.48 | 10685.21 | 9603.82 |  |  |  |  |  |  |  |  |  |  |  |  |  |  |  |  |  |
|  |  | **WI** | 0.83 | 0.88 | 0.87 | 0.92 | 0.91 | 0.96 | 0.91 | 0.94 |  |  |  |  |  |  |  |  |  |  |  |  |  |  |  |  |  |
|  |  | **LMI** | 0.45 | 0.50 | 0.46 | 0.52 | 0.50 | 0.65 | 0.52 | 0.55 |  |  |  |  |  |  |  |  |  |  |  |  |  |  |  |  |  |
|  |  | **R2** | 0.56 | 0.67 | 0.67 | 0.76 | 0.74 | 0.86 | 0.74 | 0.79 |  |  | | | | | | | | | | | | | | | |
|  | **2016** | **RMSE** | 8738.14 | 6837.50 | 7116.51 | 5752.76 | 7052.87 | 5400.94 | 6741.70 | 5134.78 |  |  | | | | | | | | | | | | | | | |
|  |  | **WI** | 0.91 | 0.95 | 0.94 | 0.97 | 0.95 | 0.97 | 0.96 | 0.97 |  |  | | | | | | | | | | | | | | | |
|  |  | **LMI** | 0.52 | 0.60 | 0.60 | 0.66 | 0.58 | 0.70 | 0.57 | 0.69 |  |  | | | | | | | | | | | | | | | |
|  |  | **R2** | 0.69 | 0.81 | 0.80 | 0.87 | 0.80 | 0.88 | 0.82 | 0.89 |  |  | | | | | | | | | | | | | | | |
| **Stacked LSTM** | **1998** | **RMSE** | 15392.32 | 10913.63 | 13839.18 | 11219.90 | 13645.69 | 11542.46 | 13986.39 | 11354.88 |  |  | | | | | | | | | | | | | | | |
|  |  | **WI** | 0.80 | 0.90 | 0.83 | 0.90 | 0.85 | 0.90 | 0.84 | 0.91 |  |  | | | | | | | | | | | | | | | |
|  |  | **LMI** | 0.37 | 0.55 | 0.39 | 0.50 | 0.39 | 0.50 | 0.39 | 0.52 |  |  | | | | | | | | | | | | | | | |
|  |  | **R2** | 0.46 | 0.73 | 0.56 | 0.71 | 0.57 | 0.69 | 0.55 | 0.70 |  |  | | | | | | | | | | | | | | | |
|  | **2016** | **RMSE** | 10168.99 | 5996.88 | 9671.06 | 7012.46 | 10642.35 | 6523.28 | 8847.33 | 8042.89 |  |  | | | | | | | | | | | | | | | |
|  |  | **WI** | 0.87 | 0.96 | 0.88 | 0.95 | 0.85 | 0.96 | 0.90 | 0.92 |  |  | | | | | | | | | | | | | | | |
|  |  | **LMI** | 0.44 | 0.65 | 0.47 | 0.58 | 0.37 | 0.65 | 0.48 | 0.50 |  |  | | | | | | | | | | | | | | | |
|  |  | **R2** | 0.58 | 0.85 | 0.62 | 0.80 | 0.54 | 0.83 | 0.68 | 0.74 |  |  | | | | | | | | | | | | | | | |
|  |  |  |  |  |  |  |  |  |  |  |  |  | | | | | | | | | | | | | | | |

|  |  |  |  |  |  |  |  |  |  |  |  |  |  |  |
| --- | --- | --- | --- | --- | --- | --- | --- | --- | --- | --- | --- | --- | --- | --- |
| **Model** | **Station** | **Datong** | | | | | | | |  |  |  |  |  |
|  | **Year** | **1998 Max Flow(M^3^)/Month** | | | | **2016 Max Flow(M^3^)/Month** | | | |  |  |  |  |  |
|  | **Min Pd** | **6** | **12** | **18** | **24** | **6** | **12** | **18** | **24** |  |  |  |  |  |
|  | **Observed** | 77065.00/8 | | | | 65758.00/7 | | | |  | | | | |
| **Stacked** | **1 feature** | 57211.82/9 | 53656.70/8 | 51463.75/8 | 56766.26/8 | 51303.70/8 | 48951.63/8 | 42252.02/6 | 48679.22/8 |  | | | | |
| **LSTM** | **2 features** | 64647.34/8 | 63604.23/8 | 57249.11/8 | 58708.07/8 | 58042.62/6 | 60753.18/8 | 55705.89/6 | 53397.02/6 |  | | | | |
| **Conv En De LSTM** | **1 feature** | 55976.55/9 | 57560.53/8 | 63218.27/8 | 62804.75/8 | 53913.72/8 | 55936.09/8 | 60353.25/8 | 58593.31/8 |  | | | | |
|  | **2 features** | 60367.56/8 | 65174.24/8 | 70113.20/8 | 69740.06/9 | 58457.18/8 | 63363.18/7 | 63425.56/7 | 60643.40/7 |  | | | | |
| **Conv En De GRU** | **1 feature** | 54054.31/9 | 58217.41/9 | 59528.55/8 | 59591.22/8 | 52622.26/8 | 53630.74/7 | 57775.40/8 | 63374.66/6 |  | | | | |
|  | **2 features** | 59583.14/8 | 67916.24/8 | 73878.27/8 | 68581.81/8 | 55687.05/7 | 60457.82/7 | 60273.86/6 | 59028.74/6 |  | | | | |
|  |  |  |  |  |  |  |  |  |  |  | | | | |
|  | | | | | | | | | | | | | | |
|  |  |  |  |  |  |  |  |  |  |  |  |  |  |  |

Table 4 The peak flow prediction of Datong

|  |  |  |  |  |  |  |  |  |  |  | | | | |
| --- | --- | --- | --- | --- | --- | --- | --- | --- | --- | --- | --- | --- | --- | --- |
| **Model** | **Station** | **Hankou** | | | | | | | |  | | | | |
|  | **Year** | **1998 Max Flow(M^3^)/Month** | | | | **2016 Max Flow(M^3^)/Month** | | | |  | | | | |
|  | **Min Pd** | **6** | **12** | **18** | **24** | **6** | **12** | **18** | **24** |  | | | | |
|  | **Observed** | 67219.00/8 | | | | 49355.00/7 | | | |  | | | | |
| **Stacked** | **1 feature** | 47949.05/9 | 42226.30/8 | 38507.58/8 | 44690.96/8 | 34898.43/8 | 36758.89/8 | 31422.67/8 | 33545.99/7 |  | | | | |
| **LSTM** | **2 features** | 49315.43/9 | 47666.97/8 | 47669.21/8 | 48944.84/7 | 40608.11/7 | 40982.64/7 | 42316.09/7 | 42020.28/7 |  | | | | |
| **Conv En De LSTM** | **1 feature** | 42814.24/9 | 43659.18/9 | 47798.03/9 | 45552.51/8 | 36811.79/8 | 40672.40/6 | 41455.50/6 | 41421.19/8 |  | | | | |
|  | **2 features** | 46918.94/8 | 45675.12/8 | 57500.74/9 | 47941.18/7 | 45235.79/7 | 44817.63/7 | 48776.30/6 | 43024.21/6 |  | | | | |
| **Conv En De GRU** | **1 feature** | 42117.32/9 | 42880.25/9 | 48480.34/9 | 47258.80/9 | 36257.22/8 | 38662.68/6 | 41743.10/6 | 40968.08/8 |  | | | | |
|  | **2 features** | 47496.47/8 | 52758.74/9 | 53141.31/9 | 55788.13/9 | 44141.22/7 | 48966.08/7 | 48445.01/7 | 39724.06/6 |  | | | | |
|  | | | | | | | | | | | | | | |
|  |  |  |  |  |  |  |  |  |  |  |  |  |  |  |

Table 3 The peak flow prediction of Hankou
